# A mammalian methylation array for profiling methylation levels at conserved sequences

**DOI:** 10.1038/s41467-022-28355-z

**Published:** 2022-02-10

**Authors:** Adriana Arneson, Amin Haghani, Michael J. Thompson, Matteo Pellegrini, Soo Bin Kwon, Ha Vu, Emily Maciejewski, Mingjia Yao, Caesar Z. Li, Ake T. Lu, Marco Morselli, Liudmilla Rubbi, Bret Barnes, Kasper D. Hansen, Wanding Zhou, Charles E. Breeze, Jason Ernst, Steve Horvath

**Affiliations:** 1 Bioinformatics Interdepartmental Program, University of California, Los Angeles, CA 90095 USA; 2 Department of Biological Chemistry, University of California, Los Angeles, Los Angeles, CA USA; 3 Dept. of Human Genetics, David Geffen School of Medicine, University of California Los Angeles, Los Angeles, CA 90095 USA; 4 Molecular, Cell and Developmental Biology, University of California Los Angeles, Los Angeles, CA 90095 USA; 5 Computer Science Department, University of California, Los Angeles, Los Angeles, CA USA; 6 Dept. of Biostatistics, Fielding School of Public Health, University of California Los Angeles, Los Angeles, CA 90095 USA; 7 Illumina, Inc, 5200 Illumina Way, San Diego, CA 92122 USA; 8 Department of Biostatistics, Johns Hopkins Bloomberg School of Public Health, Baltimore, MD USA; 9 Department of Genetic Medicine, Johns Hopkins School of Medicine, Baltimore, MD USA; 10 Center for Computational and Genomic Medicine, Children’s Hospital of Philadelphia, Philadelphia, USA; 11 Altius Institute for Biomedical Sciences, Seattle, WA USA; 12 Eli and Edythe Broad Center of Regenerative Medicine and Stem Cell Research at University of California, Los Angeles, Los Angeles, CA USA; 13 Department of Computational Medicine, University of California, Los Angeles, Los Angeles, CA USA; 14 Jonsson Comprehensive Cancer Center, University of California, Los Angeles, Los Angeles, CA USA; 15 Molecular Biology Institute, University of California, Los Angeles, Los Angeles, CA USA; 16 Altos Labs, San Diego, CA USA

**Keywords:** DNA methylation, Data acquisition, Molecular evolution

## Abstract

Infinium methylation arrays are not available for the vast majority of non-human mammals. Moreover, even if species-specific arrays were available, probe differences between them would confound cross-species comparisons. To address these challenges, we developed the mammalian methylation array, a single custom array that measures up to 36k CpGs per species that are well conserved across many mammalian species. We designed a set of probes that can tolerate specific cross-species mutations. We annotate the array in over 200 species and report CpG island status and chromatin states in select species. Calibration experiments demonstrate the high fidelity in humans, rats, and mice. The mammalian methylation array has several strengths: it applies to all mammalian species even those that have not yet been sequenced, it provides deep coverage of conserved cytosines facilitating the development of epigenetic biomarkers, and it increases the probability that biological insights gained in one species will translate to others.

## Abstract (web summary)

Methods to probe DNA methylation in the majority of non-human mammals are lacking. Here the authors developed a Mammalian Methylation Array that includes 36k well-conserved CpGs in mammals which will facilitate cross-species comparisons. They annotate the conserved CpGs in > 200 species. The array allows one to measure methylation in all mammalian species including unsequenced ones.

## Introduction

Methylation of DNA by the attachment of a methyl group to cytosines is one of the most widely studied epigenetic modifications in vertebrates, due to its implications in regulating gene expression across many biological processes including disease^1^. A variety of different assays have been proposed for measuring DNA methylation including microarray-based methylation arrays^2,3^ and sequencing-based assays such as whole-genome bisulfite sequencing (WGBS), reduced representation bisulfite sequencing (RRBS)^4^, and targeted bisulfite sequencing^5^. Despite the availability of sequencing-based assays, array-based technology remains widely used for measuring DNA methylation due to its combination of low cost, ease of use, and high reproducibility and reliability^6^.

The first human methylation array (Illumina Infinium 27K) was introduced by Illumina Inc in 2009, which was followed by the 450K^2^ and EPIC arrays with larger coverage^6^. More recently, Illumina released a mouse methylation array (Infinium Mouse Methylation BeadChip) that profiles over 285k markers across diverse murine strains. It will probably not be economical to develop similar methylation arrays for less frequently studied mammalian species (e.g., elephants or marine mammals) due to insufficient demand. Moreover, even if costs were no impediment, species-specific arrays would likely be sub-optimal in comparative studies across different species as the measurement platforms would be different.

To address these challenges, we developed a single mammalian methylation array designed to be used to measure DNA methylation across mammals. The array targets CpGs for which the CpG and flanking sequence are highly conserved across many mammals so that the methylation of many of these CpGs can be measured in each mammal. A unique aspect of the array design is that it repurposes the degenerate base technology (originally used by Illumina Infinium probes to tolerate within-human variation) to tolerate cross-species mutations across mammalian species. To select the specific probe sequences including tolerated mutations that appear on the array we developed the Conserved Methylation Array Probe Selector (CMAPS). CMAPS takes as input a multiple sequence alignment to a reference genome and a set of probe design constraints, and selects a set of probe sequences including tolerated mutations, which can be used to query methylation in many species. We apply CMAPS to select over 35 thousand CpGs for the mammalian methylation array, which we complemented with close to two thousand known human biomarker CpGs. We characterize the CpGs on the mammalian methylation array with various genomic annotations. Further, we use calibration data to evaluate the fidelity of individual probes in humans, mice, and rats. CMAPS has led to the design of the mammalian methylation array, which will facilitate the study of cytosine methylation at conserved loci across all mammal species.

## Results

### Designing the mammalian methylation array

The CMAPS algorithm is designed to select a set of Illumina Infinium array probes such that for a target set of species many probes are expected to work in each species (see “Methods” section). Array probes are sequences of length 50 bp flanking a target CpG based on the human reference genome. Selecting sequences present in the human reference genome increases the likelihood that measurements in other species will transfer to human. The mammalian methylation array adapts the degenerate base technology for tolerating human SNPs so that probes can tolerate a limited number of cross-species mutations. The CMAPS algorithm is provided as input a multiple-species sequence alignment to a reference genome. CMAPS uses these inputs to then select the CpGs to target on the array. As part of selecting the CpGs, CMAPS also selects the probe sequence design to target them including the specific set of degenerate bases. For designing the mammalian methylation array, CMAPS was applied to the subset of 62 mammals within a 100-way alignment of 99 vertebrate genomes with the human genome^7^, but we note the CMAPS method is general.

**Fig. 1:**
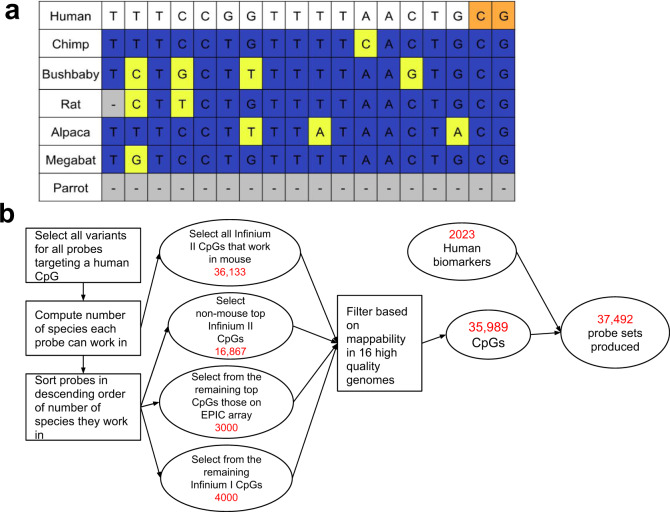
Overview of mammalian methylation array design process. **a** Toy example of a multiple sequence alignment at a CpG site being considered by the CMAPS algorithm. The orange coloring highlights the CpG being targeted. Positions, where other species have alignment that matches the human sequence, are in dark blue; positions, where other species have alignment that does not match the human sequence, are in neon yellow; positions, where other species have no alignment, are in gray. **b** Flowchart detailing the selection of probes on the array by the CMAPS algorithm. A small fraction of probes designed were dropped during the manufacturing process. The number of selected CpGs in different sets were determined by biological considerations (e.g., sufficient numbers of Type I probes to capture CpG rich regions), statistical considerations (sufficient numbers of Type I probes for normalization methods), and costs of the resulting array (fewer than 40 K CpGs resulted in tolerable costs and Type II probes being more cost-effective than Type I probes).

In designing a probe for a CpG, CMAPS considers multiple different options. One option is the type of probe. Illumina’s current methylation array technology allows up to two types of probes: Infinium I and Infinium II. The latter is newer technology requiring only one silica bead to query the methylation of a CpG, while the former requires two beads. By only requiring one bead Infinium II probes allow under fixed array capacity limits interrogating more CpGs, though Infinium I probes are better able to query CpGs in CpG rich regions^3^. Another option for each of these two types of probes is whether the probe sequence is on the forward or reverse genomic strand, giving four total combinations of options for probe type and strand for each CpG. In addition, CMAPS has options for the position and nucleotide identity of tolerated mutations. The array degenerate base technology allows for potentially up to three degenerate bases per probe sequence, which are combinations of a position and alternative nucleotide from the reference sequence that the array detection can tolerate in the sequence being interrogated. For some probes, fewer than three degenerate bases could be designed, which was determined based on a design score computed by Illumina for each probe and in the case of Infinium II probes also the number of CpGs within the probe sequence. CMAPS uses a greedy algorithm to select the tolerated mutations for each combination of probe type and strand. The algorithm aims to maximize the number of species in the alignment the probe is expected to work in based on just local alignment information that is without considering how uniquely mappable the probe is across the genome. A probe for a CpG is expected to work in a non-human species based on local alignment information if there are no differences in the alignment between the human genome sequence and the other species excluding those accounted for by the probe’s degenerate bases (Fig. 1a and see “Methods” section). For each CpG site in the human genome, CMAPS retained for further consideration the Infinium I probe out of the two options (forward or reverse of the CpG) which had the greater number of species for which the probe was expected to work, and likewise for Infinium II.

We next applied a series of rules to identify a reduced subset of candidate probes. First, we included all 36,133 Infinium II probes that were expected to work in mouse (based on the mm10 genome), which maximizes the expected array utility for one of the most widely used model organisms. For the remaining set of CpG sites not corresponding to probes selected in the previous step, we sorted them in descending order of the number of species for which an Infinium II probe was expected to work. We then added the Infinium II probes for the top 16,867 CpG sites for a total of 53,000 CpG sites. Next, we ranked the CpG sites targeted on the Illumina EPIC array^6^ in descending order of the number of species for which a probe targeting the CpG is expected to work. For this, we required the probe to be of the same probe type and strand as on the EPIC array, but used the degenerate bases picked by the CMAPS algorithm. The probe was allowed to differ in terms of degenerate base positions, as EPIC probes typically do not account for degenerate bases across species. For this, we selected the probes corresponding to the top 3000 ranked sites that had not already been picked based on the earlier criteria. CpGs that are present both on the EPIC and the mammalian array is expected to facilitate data integration with existing EPIC data from human epidemiological cohorts.

Lastly, we sorted the CpG sites in descending order of number of species for which an Infinium I probe is expected to work and picked the corresponding Infinium I probes for the top 4000 CpGs that had not already been included. We picked fewer Infinium I probes than Infinium II probes so as to be able to interrogate more CpGs at the same cost. However, we included some Infinium I probes as they also have some distinct advantages. First, type I probes allow enhanced querying of CpG dense regions such as CpG islands, as CpGs do not count towards the limited number of positions of variation as for Infinium II probes. Second, type I probes are needed for the normalization methods described below (see “Methods” section).

Overall, these selection criteria resulted in probes targeting 60,000 CpGs (Fig. 1b). For some of these 60,000 CpGs, the sequence of the probe targeting it can map to multiple locations in a genome, which could result in a confounded signal coming from multiple CpG sites. This issue is compounded by individual probes corresponding to multiple sequences reflecting different possible combinations of the degenerate bases. To identify a subset of probes less susceptible to such confounders, for 16 high-quality genomes, we computed for each probe how many of its versions map uniquely in that genome (see Methods). We performed the mapping step only for our final set of candidate probe sequences since it depends on the exact design of the probe (choice of type I versus type II, forward versus reverse strand, and position of degenerate bases). We then filtered CpGs down by requiring all versions of a probe targeting it map uniquely in at least 80% of the species they are expected to target out of the 16 high-quality genomes, unless the probe is expected to target at least 40 mammals from the alignment, in which case the mapping criterion was discarded. This reduced the set of candidate probes to targeting 35,989 CpGs.

We selected an additional 2023 probes targeting cytosine methylation array based on their utility for human biomarker studies (Supplementary Data 1). These probes, which were previously implemented in human Illumina Infinium arrays (EPIC, 450K, 27K), were selected due to their utility for human biomarker studies estimating age, blood cell counts, or the proportion of neurons in brain tissue^8–14^. The final manufactured mammalian methylation array measures cytosine levels of 37,449 unique cytosines: 37,445 of these cytosines are followed by a guanine (CpGs), of which 43 were measured by two sets of probes, and four are followed by another nucleotide (non-CpGs) giving 37,492 total probe sets. The total number of CpGs included on the array was constrained by cost considerations. The human biomarker probes included on the array included the four targeting non-CpGs and an additional 1982 targeting CpGs of which 29 also had a separate set of probes based on conservation criteria. In addition, the array contains a set of control probes used for assessing bisulfite conversion efficiency and other quality metrics. A detailed analysis of the Infinium probe context of the mammalian array and relation to human and mouse arrays is presented in Supplementary Fig. 1. The mammalian methylation array’s focus on highly conserved regions led to an array that is distinct from other currently available Infinium arrays that focus on specific species. For example, the mammalian array only shares 3107 CpGs with the Illumina Mouse Methylation array and only 7111 CpGs with the Illumina EPIC array.

### Mappability analysis in mammals

All 37,488 probe sets targeting CpGs profiled on the mammalian methylation array apply to humans, but only a subset of these applies to other species. When conducting analyses in a specific species it can thus be desirable to restrict analyses to the subset of CpGs that apply to that species. The alignment of the probes to the target genome can identify the subset of CpGs that apply to a species. In addition, the detection *p*-value can further filter out the low-quality probes. Furthermore, detection *p*-values filtering can be used even if there is no genome assembly available for the species.

**Fig. 2:**
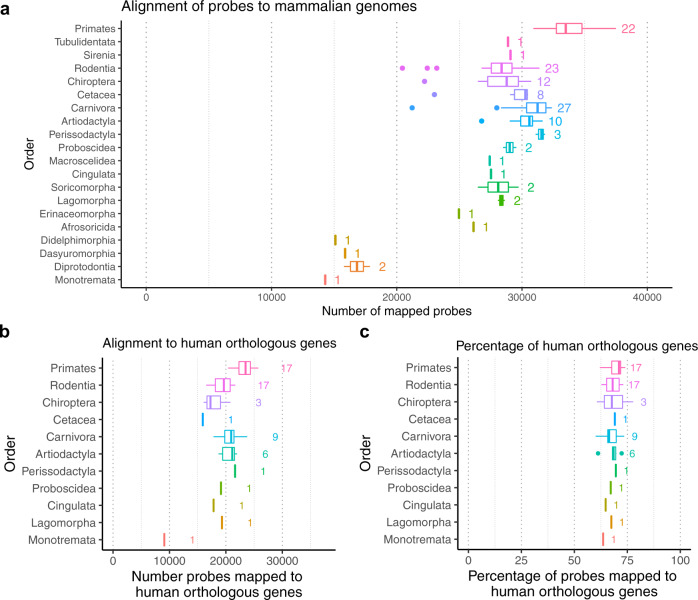
CpG and gene coverage of probes on the mammalian methylation array across different phylogenetic orders. **a** Probe localization based on the QuasR package^43^. The rows correspond to different phylogenetic orders. The phylogenetic orders are ordered based on the phylogenetic tree and increasing distance to human. The *x* axis reports the median number of mapped probes across species from the given phylogenetic order. The number to the right of each boxplot reports the number of species per order, e.g., *n* = 22 primate species. **b** The number of probes mapped to human orthologous genes for the subset of genomes in the Ensembl database (*x* axis). *n* = 17 genomes were used for primates. **c** Percentage of the probes associated with human orthologous genes among mapped probes for the species in **b**. The boxplot visualizes the median (vertical line in box) and upper and lower quartiles (25th and 75th percentile). The whiskers represent at most the 1.5*interquartile range of each order by extending to the most extreme data point that is no more than 1.5 times the interquartile range from the box.

We have mapped the array CpGs to 159 mammalian species based on the probe sequences targeting them, which provides a candidate position from which a gene for the CpG can also be associated. As expected, the closer a species is to humans, the more CpGs map to the genome of this species. Around 30k CpGs on the array map to most placental mammals (eutherians, Fig. 2a, and Supplementary Data 2). Roughly 15K CpGs map to most non-placental mammalian genomes (marsupial orders: Didelphimorpha, Dasyuromorphia, Diprotodontia), such as kangaroos or opossums. Only 14,283 CpGs map to platypus, which is an egg-laying mammal (monotreme) (Fig. 2).

A CpG that is adjacent to a given gene in humans may not map to a position adjacent to the corresponding (orthologous) gene in another species. Between 15k to 22k CpGs (~70% mapped CpGs) were assigned to human orthologous genes based on their mapped position in most phylogenetic orders (rodents, bats, carnivores, Fig. 2b, c and Supplementary Data 3).

These numbers surrounding orthologous genes are probably overly conservative (i.e., lower than the true numbers) because we found the majority of CpGs (about 58%) that do not map to orthologous genes in the non-human species are located in intergenic regions outside of promoters (see “Methods” section), which suggests that frequently at least one of the gene assignments was inaccurate.

### Chromosome and gene region coverage of array

We analyzed the chromosome and gene region coverage of the mammalian methylation array for human and mouse. The mammalian methylation has substantial coverage of all chromosomes (235–3938 and 687–3179 probes per chromosome for human and mouse, respectively), with the exception of the Y chromosome, which only has two probes in both species (Supplementary Fig. 2a). Around 80% of the probes are either in a gene body or its promoter region (Supplementary Fig. 2b). The distribution of gene region and the distances to transcriptional start sites (TSSs) are comparable between human and mouse (Supplementary Fig. 2c, d). CpGs on the mammalian array cover 6871 human and 5659 mouse genes when each CpGs is assigned uniquely to its closest gene neighbor. The gene coverage is uneven: while on average a gene is covered by 2 CpGs some genes are covered by as many as 150 CpGs. In mouse, 73% of CpGs (21,664) were assigned to a human orthologous genes (Supplementary Fig. 2e), suggesting many CpG measurements from the array in mice will be informative to humans (and vice versa).

### Gene sets represented in mammalian array

We analyzed gene set enrichments of all genes that are represented on the mammalian array using GREAT^15^. Significant gene sets are implicated in development, growth, transcriptional regulation, metabolism, cancer, mortality, aging, and survival (Supplementary Fig. 3). We also used the *TissueEnrich*^16^ software to analyze gene expression (see “Methods” section). The majority of mammalian methylation array probes (~65%) are adjacent to genes that do not exhibit clear tissue specificity in considered human and mouse tissues (Supplementary Fig. 4a, b). However, the mammalian array also contains CpGs that are adjacent to genes that are expressed in a tissue-specific manner, notably testis and cerebral cortex (Supplementary Fig. 4c).

### CpG island and methylation status

**Fig. 3:**
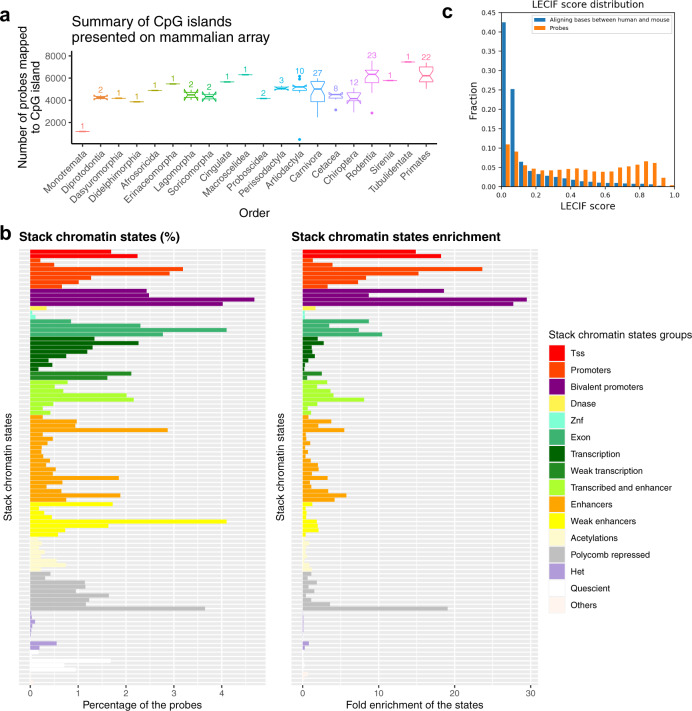
CpG island and chromatin state analysis of mammalian methylation probes. We characterize the CpGs located on the mammalian methylation array regarding **a** CpG island status in different phylogenetic orders, **b** chromatin state analysis, and **c** Learning Evidence of Conservation from Integrated Functional genomic annotations (LECIF) score of evidence of human-mouse conservation at the functional genomics level^26^. **a** Each boxplot depicts the median number of CpGs that map to CpG islands in mammalian species of a given phylogenetic order (*x* axis). The lower and upper bound of each box visualizes the lower and upper quartile of the distribution. The notch around the median number of CpGs (horizontal line inside box) depicts the 95% confidence interval. The whiskers extend to the most extreme data point, that is, no more than 1.5 times the interquartile range from the box. The numbers above each box report the number of analyzed species in each order, e.g., *n* = 22 primate species. **b** Mammalian methylation array enrichment for universal chromatin state annotations. (Left) Distribution of probe overlap with a universal chromatin state annotation by the stacked modeling approach of ChromHMM applied to data from more than 100 cell or tissue types^19^. Bars are colored based on their state corresponding state group as indicated by the legend on right. (Right) The same as left, but showing the fold enrichments of the state relative to a uniform background. The strongest enrichment is seen for some bivalent promoter states. A version of the figure with individual states labeled can be found in Supplementary Fig. 6. TSS, transcriptional start site; DNase, DNase I hypersensitivity; znf, zinc finger genes; Het, heterochromatin. **c** Comparison of distribution of LECIF score for probes on the array (orange) and aligning bases between human and mouse (blue). The LECIF score has been binned as shown on the *x* axis, and the fraction of probes or aligning bases with scores in that bin are shown on the *y* axis.

We analyzed the CpG island and DNA methylation properties of CpGs on the mammalian array. An average of 5563 (19%) of probes in the mammalian array are located in CpG islands per species based on an analysis of 143 mammalian species (Fig. 3a). We used a CpG island detection algorithm (gCluster software^17^) to determine CpG island status (Supplementary Data 4). We also analyzed human DNA methylation levels for fractional methylation called from whole-genome bisulfite sequencing data across 37 human tissues^18^ (Supplementary Fig. 5). This confirmed that the mammalian methylation array target CpGs across a wide range of fractional methylation levels.

### Chromatin and conservation state annotation

We annotated the mammalian probes with a universal chromatin state annotation, which provides a single annotation to the genome per position based on epigenomic data from more than 100 human cell and tissue types^19^ (Fig. 3b and Supplementary Fig. 6b). The mammalian methylation array had the strongest enrichments with CpGs for specific states that locate to TSSs, promoter flanking regions, bivalent promoters, or polycomb repressed regions (Fig. 3b). A separate analysis of 25 human chromatin states for 127 cells and tissues^20,21^ showed that most per cell or tissue type chromatin state annotations are represented on the mammalian methylation array but at different degrees (Supplementary Fig. 6a). Among enhancers, CpGs had greater overlap with brain and neurosphere than other tissue groups.

While the mammalian methylation array was specifically designed to profile CpGs in highly conserved stretches of DNA based on sequence conservation, we assessed whether there was also evidence of conservation at the functional genomics level using human-mouse LECIF scores^22^. The human-mouse LECIF scores quantify evidence of conservation between human and mouse at the functional genomics level using chromatin state and other functional genomic annotations from both species. In general, probes on the array had higher LECIF scores than regions that align between human and mouse in general (Fig. 3c).

As expected the CpGs on the mammalian array cover genomic regions that are annotated to be highly conserved according to four annotations based on constrained sequence elements^23–26^ (Supplementary Data 5). Compared to the background of all 28 million CpGs in the human genome, the 37K mammalian CpGs had fold enrichments ranging from 10.2 to 16.4 fold for the different constrained sequence element sets. We carried out additional enrichment studies with respect to ConsHMM conservation states, which are based on the combinatorial and spatial patterns of which species align to and match the human reference genome at each nucleotide^27^. We used ConsHMM conservation state annotations of the human genome defined based on a 100-way vertebrate alignment. Only six ConsHMM conservation state annotations out of 100 states from the vertebrate alignment showed any enrichment (Supplementary Data 5). The four states which showed the strongest enrichment (11.6–37.2 fold) were all previously associated with a high frequency of mammalian and at least some non-mammalian vertebrates aligning to and matching the human reference genome^27^. These results demonstrate the large representation of conserved CpGs on the array.

### Mammalian array study of calibration data

**Fig. 4:**
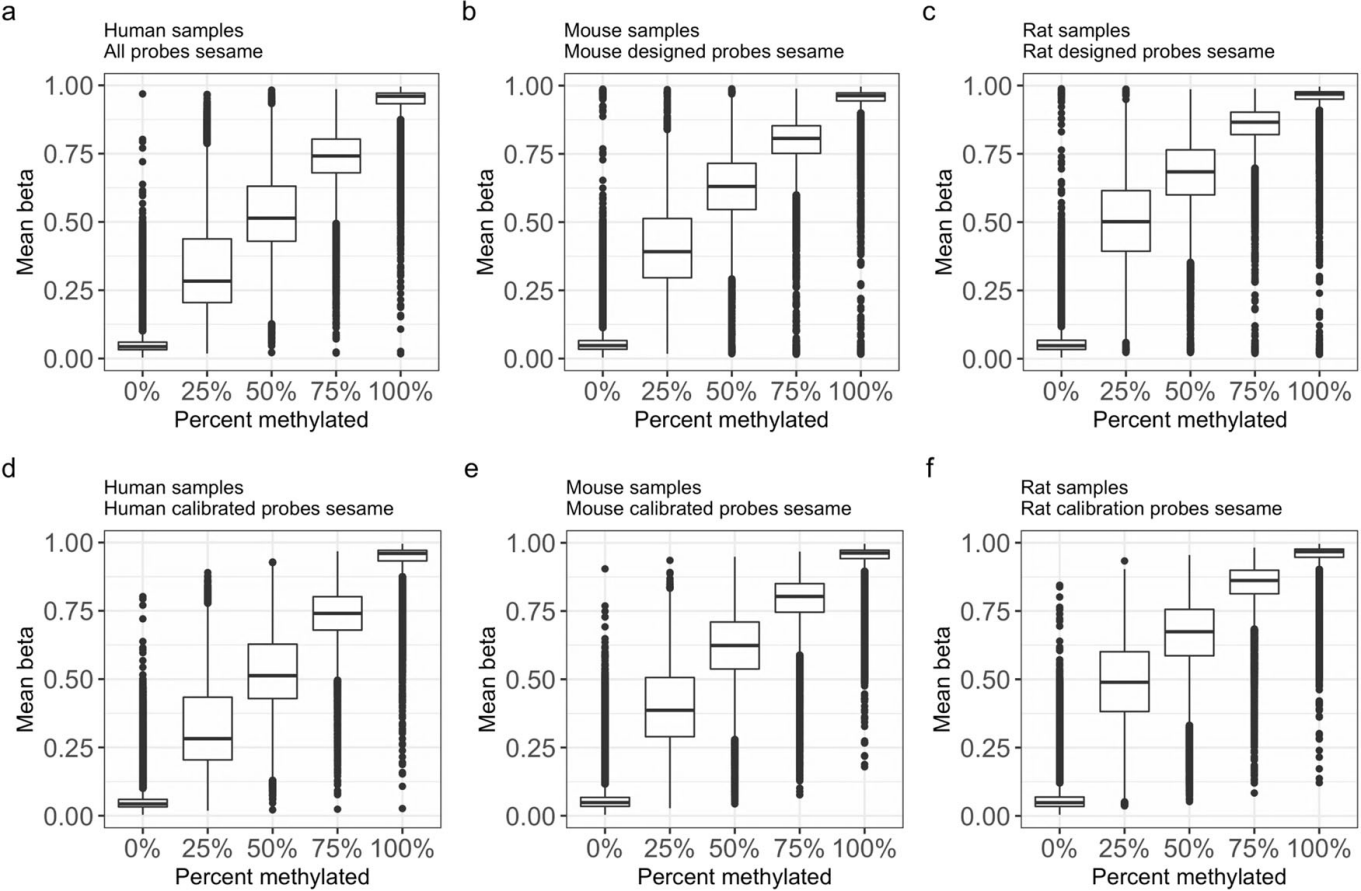
Distribution of beta values after SeSaMe normalization. **a**–**c** Distribution of beta values (relative intensity) of all probes on the array after SeSaMe normalization for **a** human samples, **b** mouse samples, and **c** rat samples. These cytosines are based on the CMAPS design criteria, i.e., **a** *n* = 35,453 human cytosines, **b** *n* = 21,900 mouse cytosines, **c** *n* = 18,157 rat cytosines. **d**–**f** Analogous to **a**–**c** but based on mappable cytosines from QuasR and after using calibration data to identify and remove severely outlying cytosines. Specifically, the lower panels use respective subsets of cytosines whose Pearson correlation with Percent methylated exceeds 0.8, which was: *n* = 37,152 CpGs for human, *n* = 27,966 for mouse, and *n* = 25,669 for rat. Beta-valued distributions are heteroscedastic in that distributions at a fractional methylation value close to 0.5 are expected to have a higher variance than those at fractional value close to zero or 1. Based on the binomial distribution, one would expect that the variance and mean value across of the SeSaMe normalized beta values across designed CpGs follow the following relationship: variance = constant*mean*(1 − mean). Indeed, in a separate analysis, we find that the left-hand side (variance) is highly correlated with the mean*(1 − mean) in mice (Pearson correlation *r* = 0.92), rats (*r* = 0.95), and humans (*r* = 0.86). It can be advisable to use statistical models and distributions that model the over-dispersion inherent in these data. Both array and sequencing methods that use bisulfite conversion followed by amplification can lead to biases in the ratio of converted to unconverted strands (beta values)^67^, which could explain the broad peaks we see in the estimate of calibration data. Each boxplot visualizes the median value and the upper and lower quartile. The whiskers extend to the most extreme data point, that is, no more than 1.5 times the interquartile range from the box.

To validate the accuracy of the mammalian methylation array we applied it to synthetic DNA methylation samples for three species: human (*n* = 10 arrays), mouse (*n* = 20), and rat (*n* = 15), where the methylation levels were known. The DNA samples from human, mouse, and rat were engineered such that the fractional methylation at all CpG sites in their genomes were ~0%, 25%, 50%, 75%, and 100% (see “Methods” section). The calibration data thus allow us to define a benchmark annotation measure, ProportionMethylated, with ordinal values 0, 0.25, 0.5, 0.75, 1. After applying the SeSaMe normalization package^28^ and subsequently removing the CpGs that were not designed to map to that species, we find that the beta values of the probes are roughly centered around the benchmark measure (ProportionMethylated) in humans, mice, and rats (Fig. 4a–c).

For each species and each CpG, we computed the correlation of DNA methylation levels with the benchmark variable ProportionMethylated across the arrays. High positive correlations would be evidence for the accuracy of the array, which is indeed what we observe. CpGs that map to the human, mouse, and rat genome have a median Pearson correlation of *r* = 0.986 with an interquartile range of [0.96,0.99], *r* = 0.959 with IQR = [0.92,0.98], and *r* = 0.956 with IQR = [0.91,0.98] with the benchmark variable ProportionMethylated in the respective species (Supplementary Data 6). The numbers of CpGs on the mammalian array that pass a given correlation threshold (irrespective of the mappability to a given species) are reported in Table 1. A few severely outlying CpGs were removed by discarding CpGs whose correlation with the benchmark variable ProportionMethylated was below 0.8 (Fig. 4d–f). We are distributing the methylation data and results from our calibration data analysis in three species (Supplementary Data 6). These calibration results will allow users to focus on cytosines whose methylation have a high correlation with the benchmark data in human, mice, or rat.

**Table 1:** Correlating DNA methylation levels with calibration data.

		No. CpGs with cor(CpG,PropMethylated) > threshold		
		Mammal		EPIC
Species	Threshold	SeSaMe	Minfi	Minfi
Mouse	0.85	27,868	26,944	4550
	0.90	24,050	22,207	2356
	0.95	16,444	12,797	604
Rat	0.85	26,425	25,779	17,650
	0.90	22,427	20,989	6159
	0.95	15,101	12,848	819
Human	0.85	36,438	35,761	–
	0.90	34,547	33,402	–
	0.95	30,327	28,445	–

We evaluated the mammalian methylation array with two different software methods for normalization: SeSaMe and Minfi (noob normalization). The EPIC array data were only normalized with the noob normalization method in Minfi. As indicated in the first column, the DNA samples came from three species: mouse (*n* = 20 mammalian arrays; *n* = 15 EPIC arrays), rat (*n* = 15 mammalian arrays; *n* = 10 EPIC arrays), and human (*n* = 10 mammalian arrays). For each species, the artificial chromosomes exhibited on average 0%, 25%, 50%, 75%, and 100% methylation at each CpG location. Thus, the variable ProportionMethylated (with ordinal values 0, 0.25, 0.5, 0.75, 1) can be considered as a benchmark/gold standard. The table reports the number of CpGs on the array for which the Pearson correlation with the ProportionMethylation was greater than the correlation threshold (second column) based on SeSaMe (third column) and Minfi (fourth column) for the mammalian methylation array and Minfi for the EPIC array (fifth column). All CpGs on the respective array were considered, i.e., 37,942 CpGs for the mammalian array and 866k CpGs on the EPIC array. The table does not report results for EPIC combined with the Minfi/noob normalization in humans because the underlying sample size (*n* = 3) was too low (“−” denotes not available).

We also compared the SeSaMe normalization with the noob normalization that is implemented in the minfi R package^29,30^. SeSaMe slightly outperforms minfi when it comes to the number of CpGs that exceed a given correlation threshold with ProportionMethylated (Table 1).

### Comparison with the human EPIC methylation array study in calibration data

We compared the mammalian methylation array to the human EPIC methylation array, which profiles 866k CpGs in the human genome. Some of the EPIC array probes are expected to apply to the mouse and rat genomes as well^31^. To facilitate a comparison between the mammalian methylation array and the human EPIC array for non-human samples, we applied the latter to calibration data from mouse (*n* = 15 arrays) and rat (*n* = 10). The same engineered DNA methylation samples were analyzed on the human EPIC array as on the mammalian methylation array above. In particular, we were able to correlate each CpG on the EPIC array with a benchmark measure (ProportionMethylated) in mice and rats (Table 1). Only 2356 (out of 866k) CpGs on the human EPIC exceed a correlation of 0.90 with ProportionMethylated in mice. By contrast, 24,050 CpGs on the mammalian array exceed the same correlation threshold in mice. Similarly, the mammalian array outperforms the EPIC array in rats: only 6159 CpGs on the EPIC array exceed a correlation of 0.90 with ProportionMethylated compared with 22,427 CpGs on the mammalian array. The results are similar for the correlation thresholds of 0.85 and 0.95 (Table 1).

**Fig. 5:**
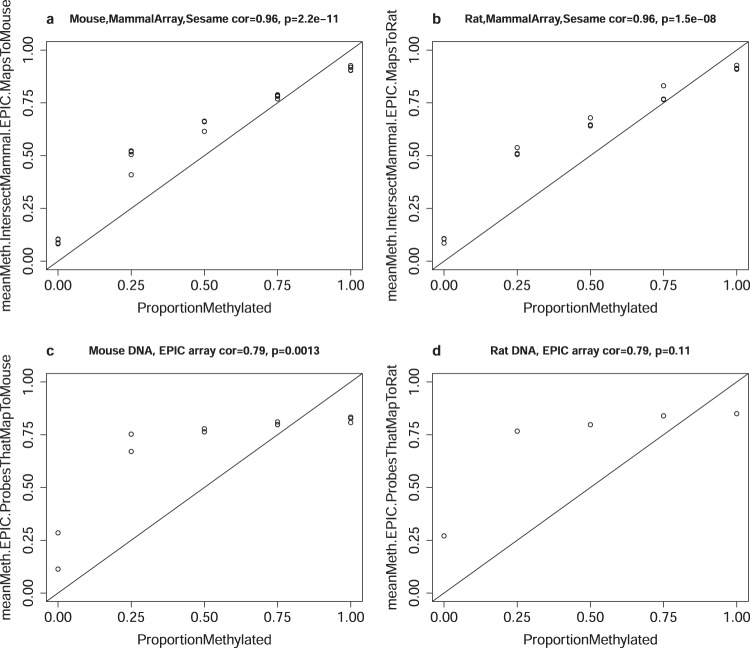
Calibration data: mean methylation across probes shared between the human EPIC array and the mammalian array. The mammalian methylation array contained 5574 probes targeting the same CpG that can also be found on the human EPIC array that was not included based on being human biomarkers. However, the mammalian array probes were engineered differently than EPIC probes so that they would more likely work across mammals. By applying both array types to calibration data, we are able to compare the calibration of the overlapping probes in mice (**a**, **c**) and rats (**b**, **d**). Upper panels (**a**, **b**) and lower panels (**c**, **d**) present the results for the mammalian array and the EPIC array, respectively. The benchmark measure (ProportionMethylated, *x* axis) versus the mean methylation value (*y* axis) across 4341 CpGs that map to mice (**a**, **c**) and 3948 CpGs that map to rats (**b**, **d**). The CpGs used to compute the mean (i) are present on the human EPIC array, (ii) present on the mammalian array, and (iii) apply to the respective species according to the mappability analysis genome coordinate file. Sample sizes: *n* = 20 arrays for mice (**a**, **c**) and *n* = 15 arrays for rats (**b**, **d**). The title reports the Pearson correlation coefficients and two-sided p-values calculated using a Student’s *t*-test.

The EPIC array contains 5574 CpGs that were also prioritized by the CMAPS algorithm based on high levels of conservation, excluding the 1986 CpGs from human biomarker studies. Out of these 5574 shared CpGs, 4341 and 3948 CpGs map to the mouse and rat genome, respectively. While human EPIC probes target the same CpG, the corresponding mammalian probe is typically different from the EPIC probe due to differences in probe type (type I versus type II probe), DNA strand, or the handling of mutations across species degenerate bases. In the following comparison, we limited the analysis to the 4341 and 3948 probes when analyzing calibration data from mice or rats, respectively. We find that the mammalian array probes are better calibrated than the corresponding EPIC array probes when applied to mouse and rat calibration data according to two different analyses that focus on shared CpGs between the two platforms. First, the mammalian array outperforms the EPIC in terms of the agreement between observed and expected mean methylation levels across the shared CpGs (*r* = 0.96 for the mammalian array and *r* = 0.79 for the EPIC array, Fig. 5). In a separate analysis, we correlated each of the shared CpGs with the benchmark value ProportionMethylated resulting in a median correlation of 0.72 for both mice and rat calibration data generated on the EPIC array. For the same probes, we observe median correlations of 0.94 and 0.93 for mice and rat calibration data generated on the mammalian array (SeSaMe normalization), respectively.

For human-to-mouse comparative DNA methylation studies, a potential alternative approach is to use the EPIC array for human samples and the mouse DNA methylation array for mouse samples and then analyze homologous CpG sites between the arrays. However, of the 286,640  CpG sites on the mouse array, we found only 14,258 sites on the mouse array aligned to the human genome and overlapped CpG sites on the EPIC array according to a liftOver analysis. A similar liftOver analysis with the 450K array instead of the EPIC array reveals only 8511 sites. In contrast, 29,637 human CpGs on the mammalian arrays also map to mouse according to a more conservative QuasR analysis of probe sequences. The mammalian array thus offers the advantages for human-mouse studies of both greater CpG coverage as well as an identical set of probe designs for the measurement.

### Comparison with RRBS and WGBS data

**Fig. 6:**
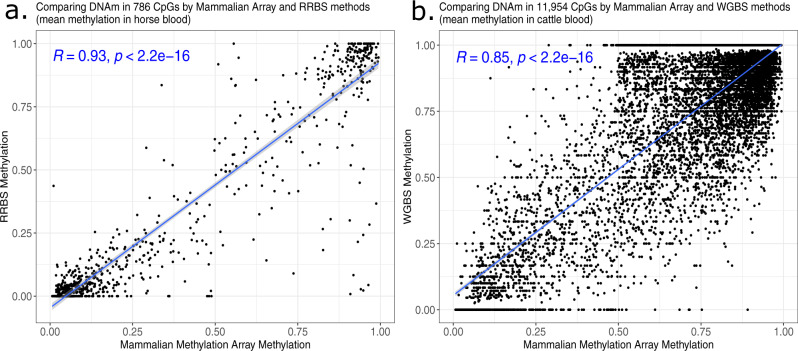
Comparison with RRBS data from horse blood and WGBS from cattle blood. Each dot corresponds to a cytosine. Mean methylation level in blood according to the mammalian array (*x* axis) versus corresponding mean values according to **a** reduced representation bisulfite sequencing and **b** whole-genome bisulfite sequencing in blood from horse and cattle, respectively. The mammalian methylation array data come from horse blood^32^ and cattle blood^33^. **a** The *y* axis reports the mean methylation levels in RRBS data from *n* = 18 whole blood samples from horses^34^. The RRBS sequence reads were downloaded from the SRA database under bioproject No. PRJNA517684 (processing described in methods). The analysis was restricted to 786 CpGs that could be mapped to both platforms. **b** mean methylation levels in WGBS data (*y* axis) from *n* = 2 blood samples from Holstein cattle^35,36^. The WGBS data are available from Gene Expression Omnibus (GSE147087). Only CpGs with sufficient read count (at least 3) were considered. The analysis was restricted to the 11,954 CpGs that could be mapped in both platforms. The blue text reports Pearson correlation coefficients and two-sided *p*-values calculated using a Student’s *t*-test. The two-sided *p*-values are at the numerical limitation of the correlation test function in R, thus capped at *p* < 2.2e−16. The blue line and shaded area correspond to a regression line and the 95% confidence interval, respectively, as determined by the default values of the R function geom_smooth.

To evaluate the agreement of mammalian methylation array data with sequencing-based data, we used mammalian methylation array data from blood samples of horses^32^ and cattle^33^ to calculate mean methylation levels for each CpG in the respective species. Next, these mean values in blood were correlated to corresponding mean values from reduced representation bisulfite sequencing (RRBS) from horses^34^ and whole-genome bisulfite sequencing data from cattle^35,36^. Even though these data sets come from different animals, from different labs, and were generated on different genomic platforms, we observed high correlations between the mean values in blood: Pearson *r* = 0.93 between horse RRBS and mammalian methylation array and *r* = 0.85 between cattle WGBS and mammalian methylation array data (Fig. 6). Overall, we find that data generated on the mammalian methylation array are highly correlated with those generated by RRBS and WGBS. These results are consistent with what was found by a separate group when correlating mammalian methylation array data with RRBS from the same 80 mouse frontal cortex DNA samples, which found a correlation of 0.79 that increased up to 0.84 when imposing specific read depth filters^37^.

### Mammalian array analysis of bats

The fact that the mammalian array applies to species whose sequence is unknown is illustrated by our large-scale study in bats that presented highly accurate epigenetic age estimators (clocks) even for bat species whose sequence is unknown^38^. Here we use the same data to illustrate that mean methylation values are highly conserved across both sequenced and non-sequenced bat species. First, we identified 21,555 CpGs that map to at least 9 different bat species according to our mappability files. For those CpGs, we calculated mean methylation levels in 16 bat species whose genome sequence was known (species *Carollia perspicillata, Desmodus rotundus, Eptesicus fuscus, Molossus molossus, Myotis brandtii, Myotis lucifugus, Myotis myotis, Nyctalus noctula, Phyllostomus discolor, Pteropus rodricensis, Pteropus vampyrus, Rhinolophus ferrumequinum, Rhynchonycteris naso, Rousettus aegyptiacus, Saccopteryx bilineata, and Tadarida brasiliensis*). The median pairwise correlation of mean methylation levels in these sequenced species was 0.88 ranging from 0.81 to 0.99. Second, we calculated mean methylation levels in 12 bat species whose genome sequence was not available at the time of this study (*Antrozous pallidus, Artibeus jamaicensis, Cynopterus brachyotis, Eidolon helvum, Leptonycteris yerbabuenae, Myotis vivesi, Nycticeius humeralis, Phyllostomus hastatus, Pteropus giganteus, Pteropus hypomelanus, Pteropus poliocephalus, and Pteropus pumilus*). In these non-sequenced species, the median pairwise correlation of mean methylation levels was 0.87 ranging from 0.79 to 0.99. Overall, these results illustrate that mean methylation levels are well-conserved between different bat species and that pairwise correlations do not depend on the sequencing status of the underlying bat species.

### Annotation for non-mammalian vertebrates

While the design of the mammalian methylation array was motivated by and only considered mammalian species, we conducted bioinformatics analysis to evaluate the expected coverage of CpGs in non-mammalian vertebrates. Specifically, we mapped the array CpGs to several non-mammalian vertebrates, including 2 fish, 3 amphibians, 45 birds, and 17 reptiles. The median number of probes that map to these species are 857 CpGs in fish (e.g., 1188 in Zebrafish), 4122 in amphibians (e.g., 5386 in Axolotl), 10,654 in birds (e.g., 11,124 in Emu; 9525 in Wild Turkey), and 10,643 in reptiles (e.g., 11,563 in Saltwater crocodile) (Supplementary Data 2). Interestingly, over 60% of these probes were aligned adjacent to human orthologous genes, which was comparable with mammals and corroborated the conservation of these probes in non-mammalian vertebrates. In contrast to mammals, only 2–14% of mappable probes (medians: 11% in fish, 2% in amphibians, 7% in birds, and 6% in reptiles) were in CpG islands. While future studies are needed to evaluate the performance of the mammalian array in non-mammalian vertebrates, our bioinformatics analysis suggests that thousands of CpGs apply to amphibians, birds, and reptiles.

## Discussion

The mammalian methylation array, which was enabled by the CMAPS algorithm for selecting conserved probes, is applicable to all mammals. Its focus on highly conserved CpGs increases the chances that findings in one species will translate to those in another species. Arrays are attractive since they facilitate high throughput operations and cost-effective measurements due to economies of scale. Our calibration data demonstrate that the array leads to high-quality measurements in three species: human, mouse, and rat. Further, the calibration data show that the mammalian methylation array greatly outperforms the human EPIC chip when it comes to high-fidelity measurements in mice and rats. The mammalian array thus is preferable for most non-human applications unless high-fidelity measurements are not needed in which case the larger content of the EPIC array may make the latter preferable.

We hypothesize that the high precision measurements of targeted CpGs on the mammalian array are due to two main reasons. First, the hybridization step of arrays enables selecting for fully bisulfite-converted DNA strands. Second, arrays provide high effective sequencing depth of specific cytosines, which is desirable for developing robust epigenetic biomarkers. Infinium arrays are widely used for DNA methylation-based biomarker studies^39^. Many users of Infinium arrays appreciate their ease of use. Many labs and core facilities already have the requisite equipment (iScan machines). Further, a large and vibrant research community of bioinformaticians has developed software pipelines for Infinium arrays.

The mammalian methylation array has several limitations. First, relatively few CpGs in a species are present on the array (tens of thousands CpGs as opposed to millions of CpGs in a given genome) and only a fraction of genes in a given species are represented by that CpGs. We briefly mention that a new expanded version of the mammalian array (denoted mammal array 320) partly addresses this limitation in mice because it combines the content of the mammalian array with that of the mouse Illumina 285K array. Second, the mammalian array focuses on CpGs in highly conserved stretches of DNA and hence does not cover parts that are specific to a given species. Third, it covers fewer CpGs in more distal species, particularly in marsupials than in placental mammals (eutherians). Finally, the calibration data suggests there are some shifts in the absolute methylation levels detected for intermediate methylation levels, but the relative order is preserved. The correct relative ordering of beta values is of primary importance in most statistical tests and analyses. Future studies should evaluate the extent the beta values measured on the mammalian array correlate with quantitative measurements from pyrosequencing, amplicon sequencing, or other measurement platforms across different species^40^. In the long run, bisulfite-free methods (e.g., EM-seq, TAPS) and other sequencing-based approaches are expected to become attractive especially as the costs of sequencing decrease and/or the robustness of these assays improve^5,36,41^.

Several software tools have been adapted for use with the mammalian methylation array that range from normalization to higher-level gene enrichment analysis. Software tools for generating normalized data adapted for use with the mammalian methylation array include SeSaMe and the minfi R package^28,29^. We expect that other normalization methods for Infinium arrays can be easily adapted for the use with the mammalian array^39,42^. The eFORGE software, which has been adapted for use with the mammalian array, facilitates chromatin state analysis and transcription factor-binding site analysis^43^. Many researchers will be interested in genome coordinates of the mammalian CpGs in different species. Toward this end, we provide genome coordinates in 159 mammalian species and 67 non-mammalian vertebrates (birds, fish, reptiles, amphibians). This list of species will increase as more high-quality genomes become available. Detailed gene annotations for CpGs in many species are available including details on gene region (e.g., exon, promoter, 5 prime untranslated region (UTR) and CpG island status (Supplementary Data 3 and Supplementary Data 7)). For human and mice, we also provide chromatin state annotations^18–20,44^ and the LECIF score on evidence of conservation at the functional genomics level between human and mouse^22^ among other annotations on our Github page^45^.

In other articles, we describe the application of the mammalian methylation array to many different mammalian species^32,38,46–52^. These studies already demonstrate that the mammalian array facilitates the development of multi-species epigenetic age estimators, which we refer to as third-generation epigenetic clocks^32,38,46–52^. The mammalian methylation array also lends itself to correlation network studies across species^53^. Overall, these applications demonstrate that the mammalian methylation array is useful for many applications.

## Methods

### Conserved Methylation Array Probe Selector (CMAPS)

Given a multi-species sequence alignment and reference genome, for each CpG site and each of the four different possible probe designs, CMAPS computes an estimate of the number of species from the alignment that could be targeted if the use of degenerate base technology is optimized for tolerated mutations. The four-probe designs involve each combination of probe type (Infinium I vs. Infinium II), and whether the probe sequence is on the forward or reverse DNA strand. For each probe option, CMAPS conducts a greedy search to select tolerated mutations, including position and allele, that maximize species coverage for the probe. The maximum number of degenerate bases that can be included in a probe is a function of a design score provided by Illumina Inc. For Infinium II probes only, CpGs present in the probe sequence count as if they are a degenerate base. More specifically, the algorithm for determining the number of species and selecting the mutations to handle performs the following steps for each probe design:

1. Let *M* be the maximum number of degenerate bases that can be designed into a specific probe, based on the design score, probe type, and CpG content.
2. For each species *s* in the alignment, let *M*_s_ be the number of mismatches in the alignment between that species and the human reference sequence of the probe
  - If *M*_s_ > *M* or the species does not have the target CpG, continue to next species.
  - If *M*_s_ ≤ *M*,
    i. For each mismatch in species *s*, add each degenerate position to a multiset *P.*
    ii. Add the species to a set *F* of feasible species to target with this probe.
3. For all |*P*| choose *M* combinations of degenerate positions of size *M* selected from *P*:
  - For each unique position in a combination *S*
    i. For each possible alternate nucleotide, count the number of species in *F* that contain that alternate nucleotide.
    ii. Pick the top *k* alternate nucleotides based on the count in *i*., where *k* is the number of occurrences of the current position in *S.*
  - Compute the number of species that match the human reference when accounting for the degenerate substitutions handled in a.
4. Select a combination of positions in *S* that maximizes 3b.

Our procedure for selecting the specific targeted CpGs and probe designs are described in the results section. We note that 29 of the CpGs selected for the mammalian methylation array based on the conservation criteria (using the sequence alignment) overlap with the human biomarker CpGs. The design of the probes targeting them could differ, however. The probe names of different probes targeting the same CpG are distinguished by extensions ‘.1’ and ‘.2’. For example, cg00350702.1 and cg00350702.2 target the same cytosine but use different probe chemistry. Probe sets targeting an additional 13 non-human biomarker CpGs and one human biomarker (cg10054641) also appeared twice on the array. The array contains four probes that measure cytosines that are not followed by a guanine, selected by human biomarkers, which are indicated with a ‘ch’ instead of a ‘cg’.

The CMAPS algorithm was applied with human hg19 as the reference genome and using the Multiz alignment of 99 vertebrates with the hg19 human genome downloaded from the UCSC Genome Browser^7,54^. For the purpose of designing the mammalian array, only the 62 mammalian species in this alignment were considered and 16 for the mappability analysis described below. However, the current version of the mappability analysis provides genome coordinates for 159 mammalian species along with 67 non-mammalian species.

The mammalian methylation array includes an additional 62 human SNP markers (whose probe names start with ‘rs’ for human studies), which can be used to detect plate map errors when dealing with multiple tissue samples collected from the same human individual. In addition, the mammalian array inherits control probes from the human EPIC array. They were composed of bisulfite conversion control, extension control, normalization, negative control, color control probes^2^. Neither control probes nor SNP markers are expected to work in non-human species.

### Mapping probes to genomic coordinates

We used two different approaches for mapping probes to genomes. The first approach (BSbolt software) was primarily used in designing the array. Subsequently, we adopted a second mappability approach (QuasR software) that allowed us to map more probes.

### Mappability approach 1: BSBolt

For version 1 of our mappability analysis (i.e., for designing the array), we applied the BSBolt mapping approach to 16 high quality genomes from: Baboon (papHam1), Cat (felCat5), Chimp (panTro4), Cow (bosTau7), Dog(canFam3), Gibbon(nomLeu3), Green Monkey (chlSab1), Horse (equCab2), Human (hg19), Macacque (macFas5), Marmoset(calJac3), Mouse (mm10), Rabbit (oryCun2), Rat (rn5), Rhesus Monkey (rheMac3), Sheep (oviAri3).

We utilized the BSBolt software^55^ package from https://github.com/NuttyLogic/BSBolt to perform the alignments. For each species’ genome sequence, BSBolt creates an in silico bisulfite-treated version of the genome. The set of nucleotide sequences of the designed probes, which includes degenerate base positions, was explicitly expanded into a larger set of nucleotide sequences representing every possible combination of those degenerate bases. For Infinium I probes, which have both a methylated and an unmethylated version of the probe sequence, only the methylated version was used as BSBolt’s version of the genome treats all CpG sites as methylated. The initial 37 K probe sequences resulted in a set of 184,352 sequences to be aligned against the various species genomes. We then ran BSBolt with parameters Align -M 0 –DB [path to bisulfite-treated genome] -BT2 bowtie2 -BT2-p 4 -BT2-k 8 -BT2-L 20 -F1 [Probe Sequence File] –O [Alignment Output File] –S to align the enlarged set of probe sequences to each prepared genome.

As we were not interested in the final BSBolt style output, we made a small modification to the code to retain its temporary output of alignment results in sam format. From these files, we collected only alignments where the entire length of the probe perfectly matched to the genome sequence (i.e., the CIGAR string 50 M and flag XM = 0). Then, for each genome we collapsed all the sequence variant alignments for each probeID down to a list of loci for that genome and for that probe.

### Mappability approach 2: QuasR

For version 2 of our mappability analysis, we aligned the probe sequences to all available mammalian genomes and 67 available non-mammalian vertebrates in ENSEMBL and NCBI Refseq databases using the QuasR package^56^. The Axolotl genome was downloaded from https://www.axolotl-omics.org website^57,58^. The fasta sequence files for each genome were downloaded from those public databases. The alignment assumed that the DNA has been subjected to a bisulfite conversion treatment. For each species’ genome sequence, QuasR creates an in silico-bisulfite-treated version of the genome. The probes were aligned to these bisulfite-treated genome sequences, which does not consider C-T as a mismatch. The alignment was ran with QuasR (a wrapper for Bowtie2) with parameters -k 2–strata–best -v 3 and bisulfite = "undir" to align the enlarged set of probe sequences to each prepared genome. From these files, we collected the best candidate unique alignment to the genome. Additionally, the estimated CpG coordinates at the end of each probe was used to extract the sequence from each genome fasta files and exclude any probes with mismatches in the target CpG location.

### Genomic loci annotations

Gene annotations (gff3) for each genome considered were also downloaded from the same sources as the genome. Following the alignment, the CpGs were annotated to genes based on the distance to the closest TSS using the Chipseeker package^59^. Genomic location of each CpG was categorized as either intergenic region, 3′ UTR, 5′ UTR, promoter (minus 10 kb to plus 100 bp from the nearest TSS), exon, or intron. The unique region assignment is prioritized as follows: exons, promoters, introns, 5′ UTR, 3′ UTR, and intergenic.

Additional genomic annotations, including human ortholog ENSEMBL IDs, were extracted for a subset of genomes with annotations available from the BioMart ENSEMBL database^60^. We compared the similarity of a candidate gene for each probe in each non-human species with human using human ortholog ENSEMBL IDs. For each probe, we examined if the assigned species ENSEMBL ID is identical to human-to-other-species-orthologous ENSEMBL ID in the human mappability (annotation) file. Orthologous comparison with human was done for genomes that could be matched to human genome by targetSpecies_homolog_associated_gene_name in Biomart using the getLDS() function.

Cell and tissue-specific chromatin state annotations were based on the 25-state ChromHMM model based on imputed data for 12-marks in human^18,21^. The universal ChromHMM chromatin state annotations that were not specific to a single human cell or tissue type were from ref. 19. The human-mouse LECIF score was from ref. 22.

To assess the coverage and enrichment of the array for a given constrained sequence element set annotation or ConsHMM conservation state, we used bedtools intersect^61^ to first determine for each CpG base if the base overlaps with the constrained element or state and if the base is included in the array. We then aggregated the results to compute the number of annotated CpG bases and the number of annotated CpG bases on the array. GERP++^26^ element annotations were downloaded from http://mendel.stanford.edu/SidowLab/downloads/gerp, PhastCons element^23^ annotations were downloaded from UCSC Table Genome Browser, and SiPhy-pi and SiPhy-omega element annotations^25^ were obtained from https://www.broadinstitute.org/mammals-models/29-mammals-project-supplementary-info. ConsHMM conservation state annotations^27^ were obtained from https://github.com/ernstlab/ConsHMM/.

### CpG island annotation

We called CpG islands using the gCluster algorithm^62^ with the default parameters. This algorithm uses clustering methods to identify the sequences that have high G + C content and CpG density. Besides CpG island status, this algorithm calculated several other attributes including length, GC content, and CpG density for each defined island. The outcome of this algorithm was a BED file that was used to annotate the probes using the annotatr package in R by checking the overlap of the aligned probes and CpG island genomic coordinates.

### Bisulfite sequencing data from the Roadmap Epigenomics Consortium

We downloaded the fraction methylated values based on whole-genome bisulfite sequencing data from 37 different cells and tissues types from the Roadmap Epigenomics Consortium (http://egg2.wustl.edu/roadmap/data/byDataType/dnamethylation/WGBS/FractionalMethylation.tar.gz)^18^. For each CpG, we averaged the fractional methylation values across the Roadmap samples.

### Reduced representation bisulfite sequencing data for horses

The raw RRBS sequence FASTA files were downloaded from the SRA database under bioproject No. PRJNA517684. However, since the processed data were not available, we realigned and processed data based on EquCab3.0.100 genome assembly. The alignment and processing of the data were done in Galaxy server with the default settings of bwa-meth^63^ and MethylDackel packages (https://github.com/dpryan79/MethylDackel). Next, we limited the analysis to the CpGs with the exact coordinates matching the horse annotations in mammalian methylation array.

### Whole-genome bisulfite sequencing data for cattle

The Bismark generated CpG reports were downloaded from the NCBI Gene Expression Omnibus under accession number GSE147087. The read mapping and DNA methylation calling were based on ARS-UCD1.2 assembly, same as the mammalian methylation array. We calculated the percent methylation at each chromosomal coordinate based on the methylated and unmethylated counts and limited the analysis to the CpGs with at least a read count of 3 and the exact coordinates matching the cattle annotations in the mammalian methylation array.

### GREAT analysis

We applied the GREAT analysis software tool^15^ to conduct gene set enrichment analysis for genes near CpGs on the array in human and mouse. The GREAT software performs both a binomial test (over genomic regions) and a hypergeometric test over genes when using a whole-genome background. We performed the enrichment based on default settings (Proximal: 5.0 kb upstream, 1.0 kb downstream, plus Distal: up to 1000 kb) for gene sets associated with GO terms, MSigDB, PANTHER, and KEGG pathway. To avoid large numbers of multiple comparisons, we restricted the analysis to the gene sets with between 10 and 3000 genes. We report nominal *p*-values and two adjustments for multiple comparisons: Bonferroni correction and the Benjamini–Hochberg false discovery rate (Supplementary Table S6).

### Tissue enrichment analysis

The enrichment of tissue-specific genes was done with the teEnrichment function in the TissueEnrich R package^16^ limited to genes and tissues in the human protein atlas^64^ and the mouse ENCODE^65^ database.

### Normalization methods

Two software scripts are currently available for extracting beta values from raw signal intensities, based on Minfi^29^ and SeSaMe^28^, respectively. Both methods use the noob method^66^ for background subtraction. For SeSaMe, the probe’s hybridization and extension performance was evaluated using Infinium-I probe out-of-band measurements (the pOOBAH method)^28^. Users can use the detection *p*-values for each CpG to filter out non-significant methylation readouts from probes unlikely to work in the target species.

### Calibration data

We generated methylation data on two different platforms: the mammalian methylation array and the human EPIC methylation array. The DNA samples from each species were enzymatically manipulated so that they would exhibit 0%, 25%, 50%, 75%, and 100% percent methylation at each CpG location, respectively. We purchased premixed DNA standards from EpigenDx Inc (products 80-8060H-PreMixHuman, 80-8060M-PreMixMouse, and Standard80-8060R-PreMixRat Premixed Calibration Standard). The variable ProportionMethylated (with ordinal values 0, 0.25, 0.5, 0.75, 1) can be interpreted as a benchmark for each CpG that maps to the respective genome. Thus, the DNA methylation levels of each CpG are expected to have a high positive correlation with ProportionMethylated across the arrays measurement from a given species. The mammalian array was applied to synthetic DNA data from 3 species: human (*n* = 10 mammalian arrays, 2 per methylation level), mouse (*n* = 20, 4 per methylation level), and rat (*n* = 15, 3 per methylation level). Similarly, the human EPIC array was applied to calibration data from mouse (*n* = 15 EPIC arrays, 3 per methylation level) and rat (*n* = 10, 2 per methylation level). The EPIC array data were normalized using the noob method (R function preprocessNoob in minfi).

### Overlap of human and mouse arrays

We aligned mouse DNA methylation array sites to the human genome (build hg19, via the UCSC liftOver tool available at https://genome.ucsc.edu/cgi-bin/hgLiftOver with minMatch = 0.1), revealing alignment for 201,461 sites. We then overlapped these aligned sites with human EPIC DNA methylation array positions and separately 450K DNA methylation array positions.

### Bat methylation analysis

For the bat methylation analysis, we used methylation data from a recent large-scale study of bat species^38^.

### Reporting summary

Further information on research design is available in the Nature Research Reporting Summary linked to this article.

## Supplementary information

Supplementary Information

Description of Additional Supplementary Data files

Supplementary Dataset 1

Supplementary Dataset 2

Supplementary Dataset 3

Supplementary Dataset 4

Supplementary Dataset 5

Supplementary Dataset 6

Supplementary Dataset 7

Reporting Summary

## Data Availability

The data that support this study are available from the corresponding authors upon reasonable request. The chip manifest file and genome annotations of the CpGs can be found on Github^45^ at https://github.com/shorvath/MammalianMethylationConsortium/tree/v1.0.0. The calibration data generated in this study have been deposited in the Gene Expression Omnibus database under accession codes GSE174567 and GSE174568. The bat methylation data are available under accession code GSE164127. The horse array data^32^ are available under accession code GSE174767. The reduced representation bisulfite sequencing from horses^34^ can be downloaded from the SRA database under bioproject No. PRJNA517684. The whole-genome bisulfite sequencing data from cattle^35,36^ can be downloaded under accession code GSE147087. The whole-genome bisulfite sequencing data from 37 different tissue types can be downloaded from the Roadmap Epigenomics Consortium^18^ at http://egg2.wustl.edu/roadmap/data/byDataType/dnamethylation/WGBS/FractionalMethylation.tar.gz. We used genome annotations from ENSEMBL [https://www.ensembl.org/index.html]. The human-mouse LECIF score^22^ can be downloaded from https://github.com/ernstlab/LECIF/. The universal ChromHMM chromatin state annotations can be downloaded from https://github.com/ernstlab/full_stack_ChromHMM_annotations. The per cell or tissue type specific chromatin state annotations in human can be downloaded from https://egg2.wustl.edu/roadmap/data/byFileType/chromhmmSegmentations/ChmmModels/imputed12marks/jointModel/final/^18,21^. The ConsHMM conservation state annotations can be downloaded from https://github.com/ernstlab/ConsHMM/^27^. The constrained element annotations can be downloaded from http://mendel.stanford.edu/SidowLab/downloads/gerp (GERP++)^26^, https://www.broadinstitute.org/mammals-models/29-mammals-project-supplementary-info (SiPhy-omega and SiPhy-omega)^25^, and https://genome.ucsc.edu/cgi-bin/hgTables (PhastCons)^23^. The cattle data generated on the mammalian array were not generated for this study. These data are presented in another article^33^ and can be requested from SH. The mammalian methylation array (HorvathMammalMethylChip40) is registered at the NCBI Gene Expression Omnibus (GEO) as platform GPL28271. The mammalian methylation array can be purchased from the non-profit Epigenetic Clock Development Foundation (https://clockfoundation.org/). A subset of annotations of the array can also be found in Supplementary Data 7. Source data are provided with this paper.

## Code availability

The CMAPS source code v1.0.0 is available from https://github.com/shorvath/MammalianMethylationConsortium/tree/v1.0.0. A vignette on using the mammalian methylation array with SeSaMe is available from https://bioconductor.org/packages/release/bioc/vignettes/sesame/inst/doc/mammal.html.
