# Supplementary material for: A mammalian methylation array for profiling methylation levels at conserved sequences
Source: Nat Commun. 2022 Feb 10;13:783. doi: 10.1038/s41467-022-28355-z (PMC8831611; doi:10.1038/s41467-022-28355-z)
Supplement: Supplementary file 1 — Supplementary Information [file 41467_2022_28355_MOESM1_ESM.pdf]

Supplementary Information

for "A mammalian methylation array for profiling methylation levels at conserved sequences" by Arneson A, Haghani A, et al

Supplementary Figures

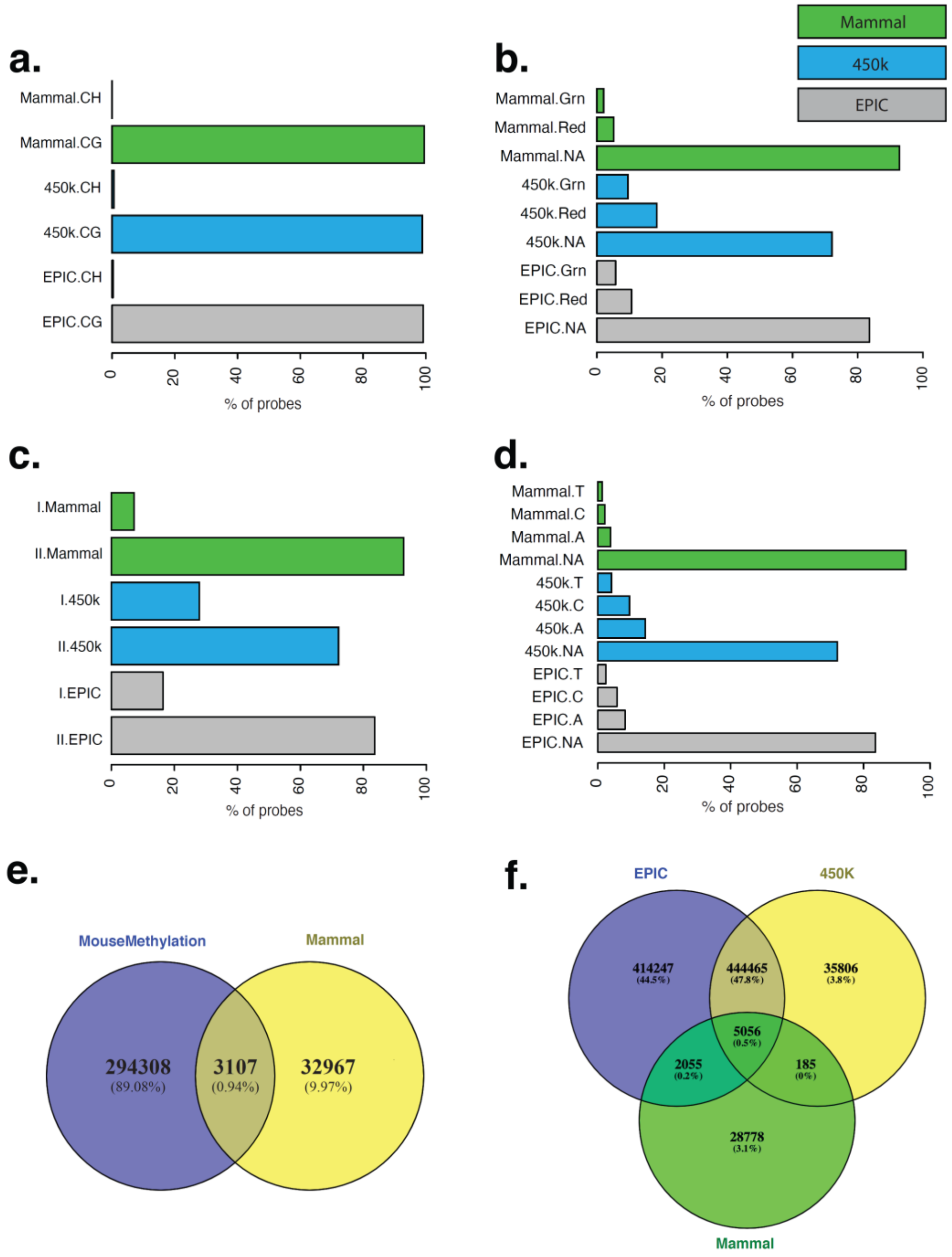

Supplementary Fig. 1: Comparison of probe context between the Illumina EPIC, 450K and the Mammalian Methylation array: (a) Analysis of CpG and non-CpG (CH) probes, **(b)** color channel assignment, **(c)** type I and type II probes, and **(d)** next base reveals similar percentages across probes from these three array platforms. Color channel assignment and probe base pair context are important for DNA methylation array analysis and the similarity between these different arrays can facilitate extension of published analysis and normalization methods. Analysis

of type I and type II probes shows a slightly lower percentage of type I probes for the mammalian methylation array. Type I probes assay DNA methylation using one color channel and two bead types, i.e. one unmethylated bead type and one methylated bead type. Conversely, type II probes assay DNA methylation using one bead type and two color channels indicating methylated and unmethylated cytosines. Adjustment for DNA methylation signal detected by these different probe types is one of the most important steps in DNA methylation array normalization, and a sufficient number of type I probes were included in the mammalian methylation array to facilitate the extension of published data normalization methods. **(e)** Comparison of shared and non-shared probes between the mammalian methylation array and the mouse methylation array loci reveals 3107 shared probes. **(f)** Comparison of shared and non-shared probes between the EPIC, 450k and the mammalian methylation array. Comparative analysis was performed using Illumina probe IDs, which are unique to each CpG. Intersection of IDs between arrays reveals over 5,000 probes that are common to all platforms (center). These probes can be used to follow up published human epigenome-wide association study (EWAS) results in model organisms such as mouse (*Mus musculus*) or rat (*Rattus norvegicus*), or across a range of other species, including all primates and other mammals.

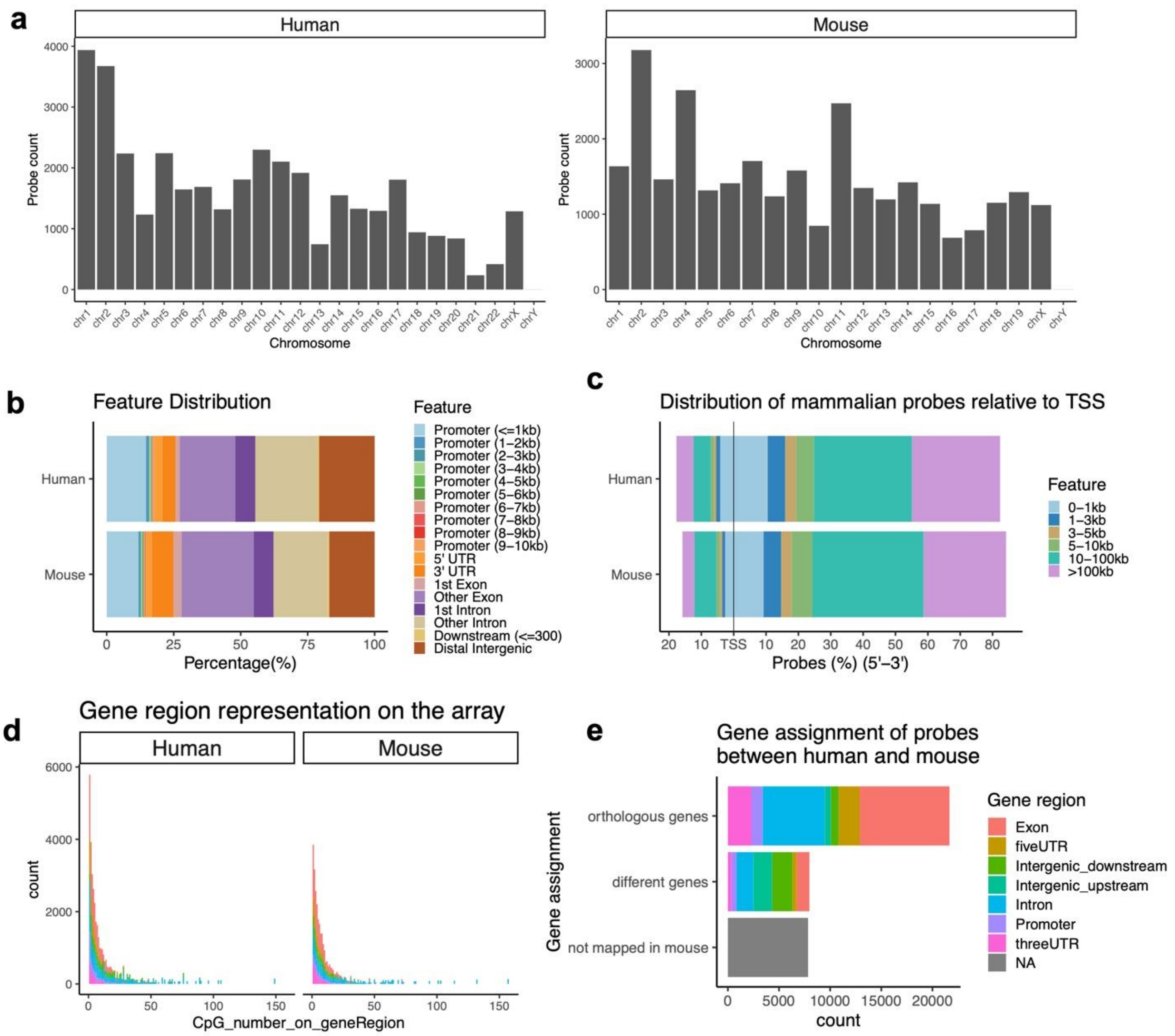

Supplementary Fig. 2. Chromosome and gene region analysis of mammalian methylation probes in humans and mice. The analysis is based on mapping probes on the mammalian methylation array to the human (hg19) and mouse (mm10) genome using the QuasR package [1]. **(a)** The number of probes per human and mouse chromosome. **(b)** The panel reports the percentage of probes that are located in different gene regions (promoters, 5' UTR, 3' UTR, introns, exons) in humans and mice. **(c)** The panel reports the distribution of the probes relative to the nearest transcriptional start site (TSS). **(d)** Histogram of CpG number in different combinations of genes and gene regions (as defined in the legend of panel e, where regions are assigned based on the closest gene) in human and mouse genomes. For example, there is a gene in human and a gene in mouse with about 150 CpGs inside its introns on the array. **(e)** Alignment to orthologous genes between humans and mice. The colors indicate the mapped gene region in the mouse genome. The unique region assignment are prioritized as follows: exons, promoters, introns, 5' UTR, 3' UTR.

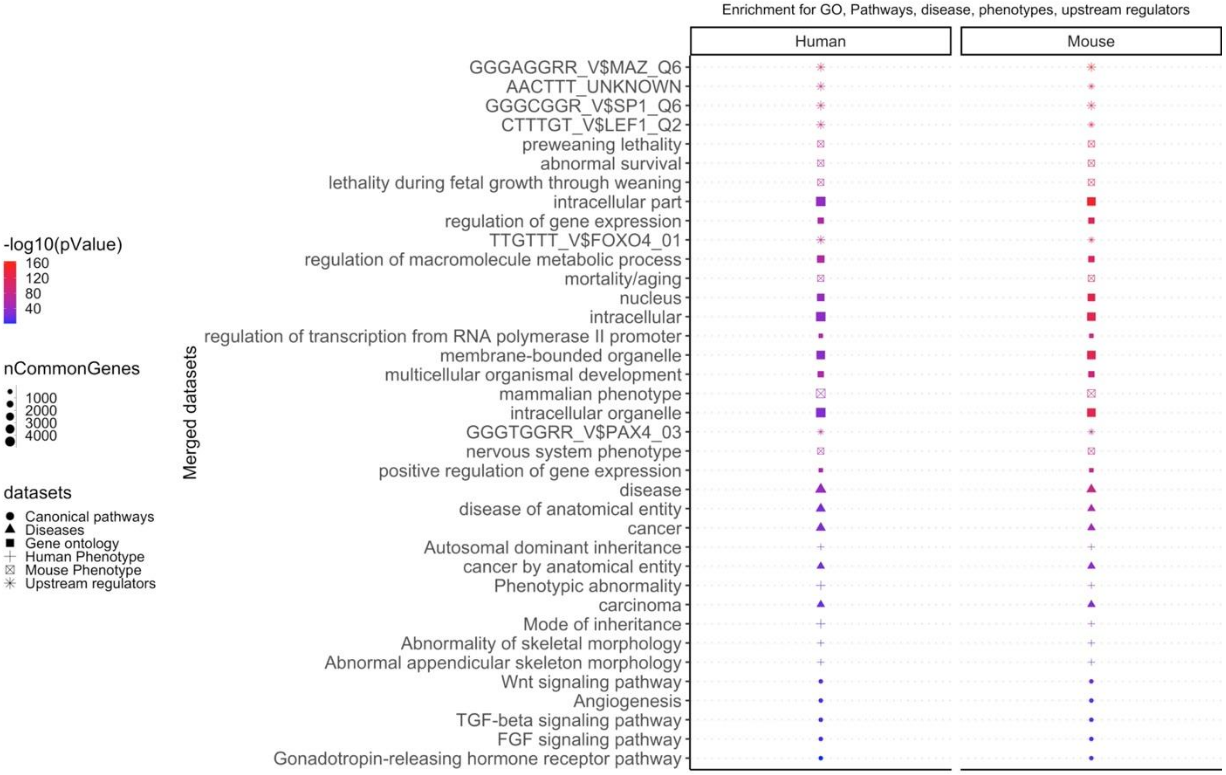

Summary Figure 3. GREAT gene set enrichment analysis of all probes on the mammalian methylation array. The figure shows the top enriched gene sets based on gene-level enrichment analysis for genes proximal to probes using hypergeometric statistical test in GREAT tool. The two columns correspond to enrichment analysis for human (hg19) and mouse (mm10) genomes, respectively, using the whole genome as background. The top five enriched datasets from each category (Canonical pathways, diseases, gene ontology, human and mouse phenotypes, and upstream regulators) were selected and further filtered for significance at $p < 10^{-5}$. The category is indicated by the shape, the number of genes by the size of the shape, and the significance of the enrichment is indicated by the color scale. The p-values are nominal two-sided p-values. Bonferroni and FDR corrected p-values can be found in Supplementary Data 6.

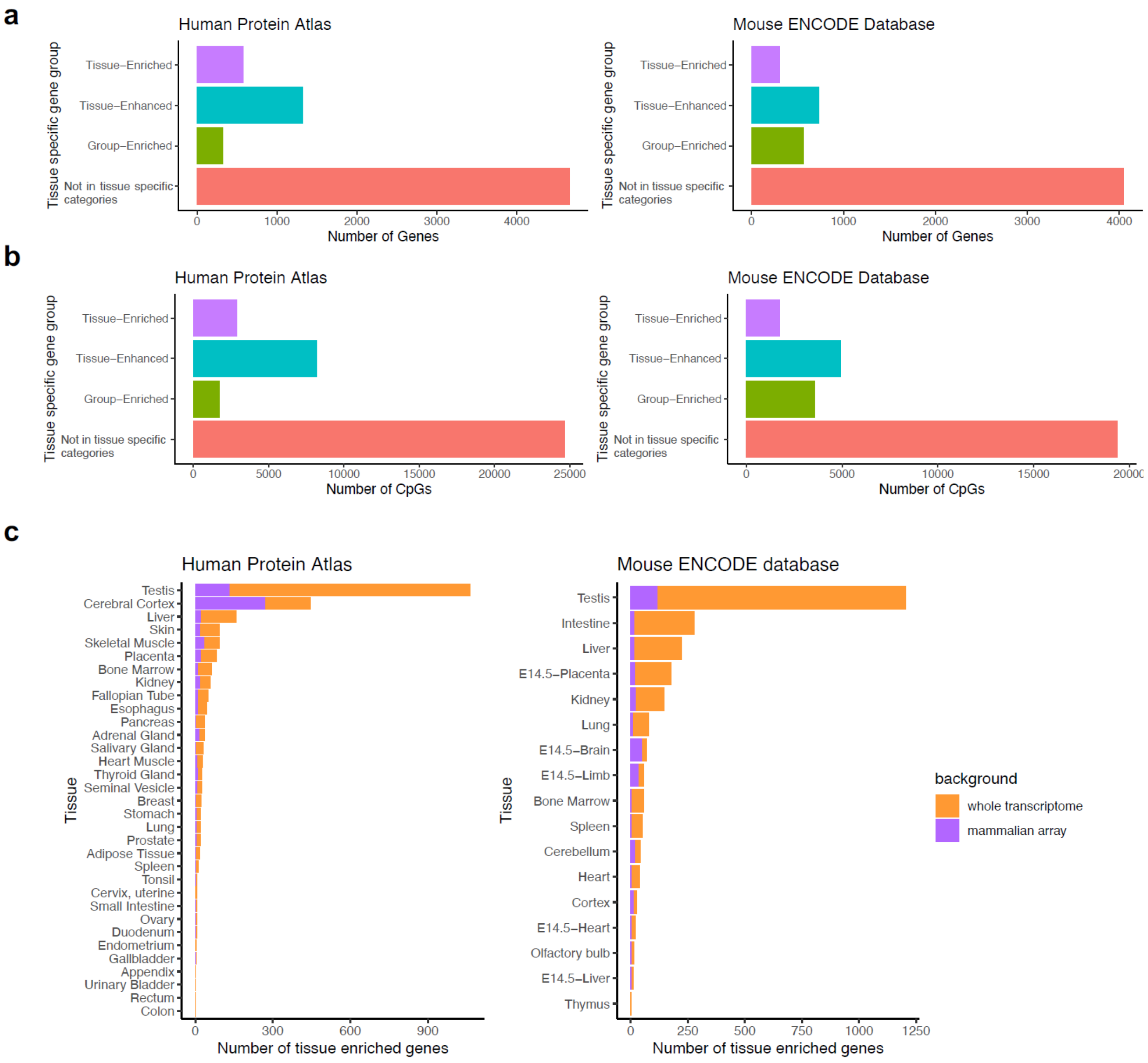

Supplementary Fig. 4. Human and mouse tissue-specific probes on mammalian methylation array. Characterization of the tissue specificity of CpG probes on the mammalian methylation array using the human protein atlas [2] and mouse ENCODE [3] databases. The left and right panels report results for human and mouse genomes, respectively. Each probe is mapped to the closest gene while other genes in the flanking region are ignored in this analysis. The number of genes **(a)** and the number of CpG probes **(b)** versus a categorical measure of tissue specificity. The categories on the y-axis have the following definitions. The following categories are defined in the "TissueEnrich" software "**Tissue Enriched"** labels genes with an expression level greater than 1 (TPM or FPKM) that also have at least five-fold higher expression levels in a particular tissue compared to all other tissues. "**Group Enriched"** labels genes with an expression level greater than 1 (TPM or FPKM) that also have at least five-fold higher expression levels in a group of 2-7 tissues compared to all other tissues, and that are not considered Tissue Enriched. **"Tissue Enhanced"** labels genes with an expression level greater than 1 (TPM or

FPKM) that also have at least five-fold higher expression levels in a particular tissue compared to the average levels in all other tissues, and that are not considered Tissue Enriched or Group Enriched. The last category displayed is defined as genes not in the **“Tissue Enriched”**, **“Group Enriched”**, or **“Tissue Enhanced”**, which includes the constitutive expressed genes. **(c)** The number of tissue-enriched genes represented on mammalian array vs background in human and mouse transcriptome.

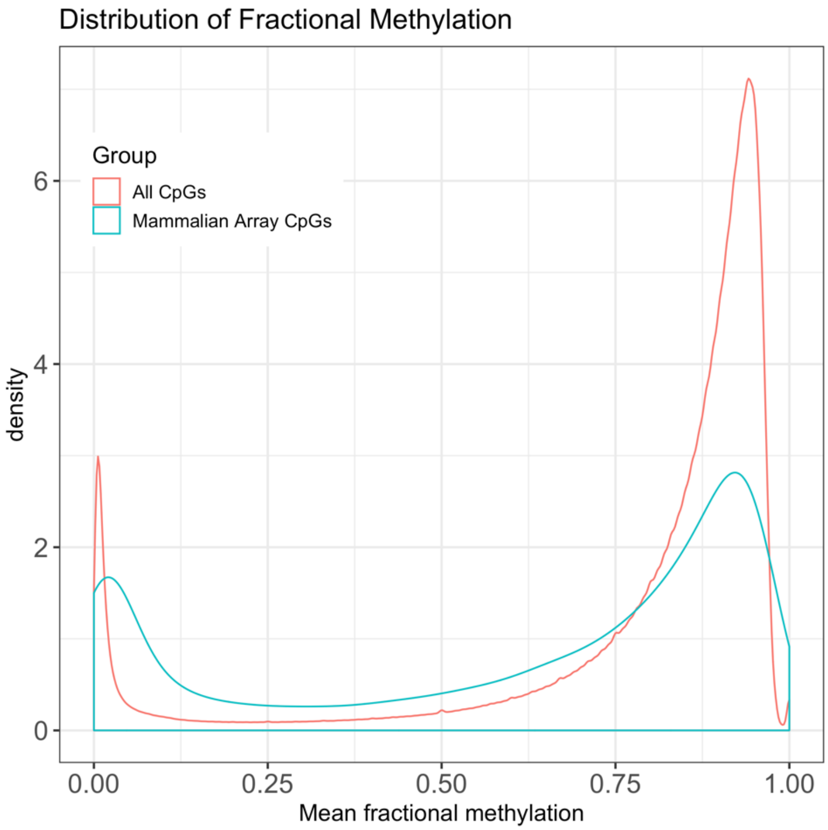

Supplementary Fig. 5. Distribution of fractional DNA methylation levels. Distribution of average fractional methylation across 37 cell and tissue types [4] at CpG sites on the array (blue) and all sites in the genome (red).

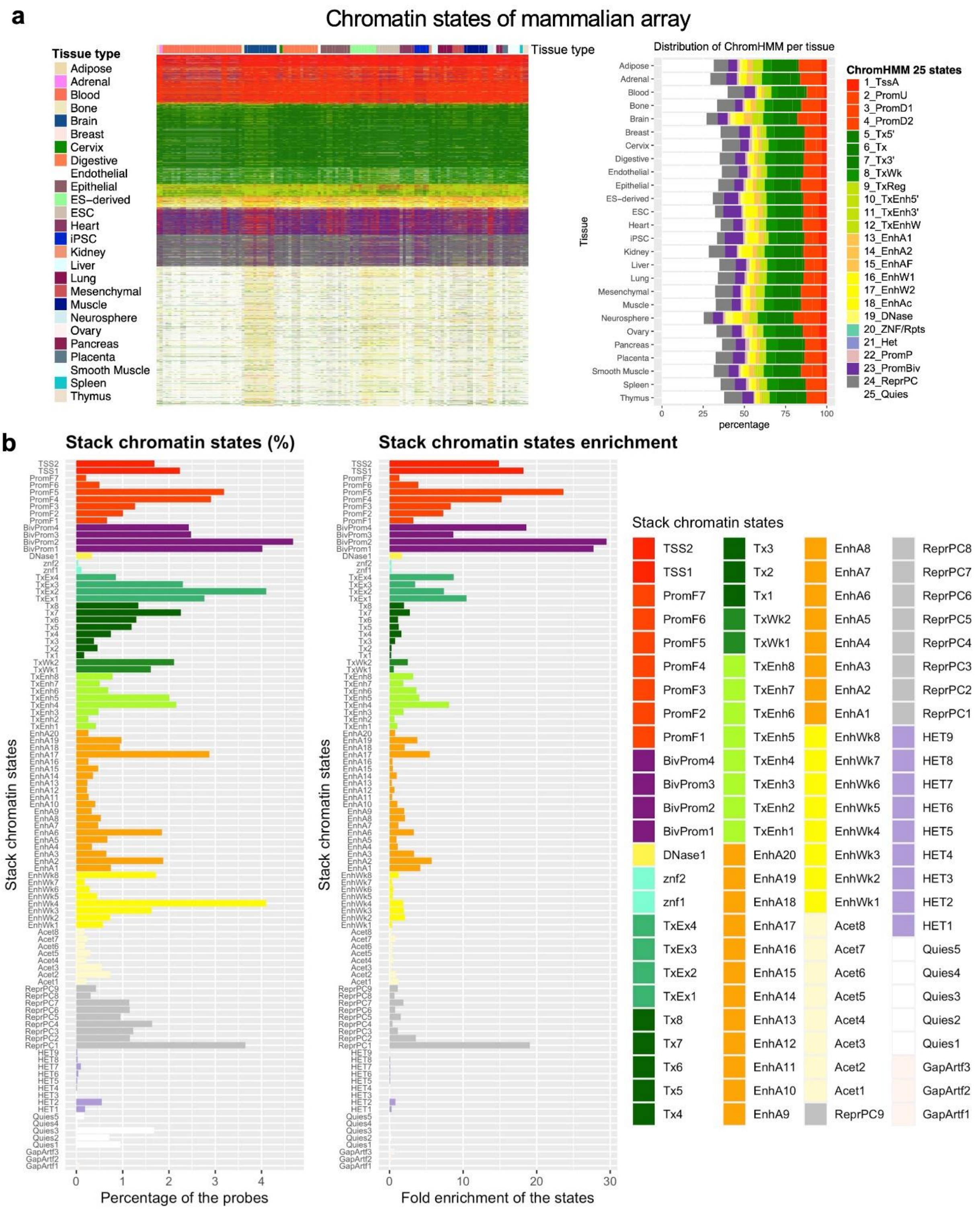
a
Chromatin states of mammalian array
Tissue type
Adipose
Adrenal
Blood
Bone
Brain
Breast
Cervix
Digestive
Endothelial
Epithelial
ES−derived
ESC
Heart
iPSC
Kidney
Liver
Lung
Mesenchymal
Muscle
Neurosphere
Ovary
Pancreas
Placenta
Smooth Muscle
Spleen
Thymus
Distribution of ChromHMM per tissue
Tissue
percentage
ChromHMM 25 states
1_TssA
2_PromU
3_PromD1
4_PromD2
5_Tx5'
6_Tx
7_Tx3'
8_TxWk
9_TxReg
10_TxEnh5'
11_TxEnh3'
12_TxEnhW
13_EnhA1
14_EnhA2
15_EnhAF
16_EnhW1
17_EnhW2
18_EnhAc
19_DNase
20_ZNF/Rpts
21_Het
22_PromP
23_PromBiv
24_ReprPC
25_Quies
b
Stack chromatin states (%)
Stack chromatin states enrichment
Stack chromatin states
Percentage of the probes
Fold enrichment of the states
Stack chromatin states
TSS2
TSS1
PromF7
PromF6
PromF5
PromF4
PromF3
PromF2
PromF1
BivProm4
BivProm3
BivProm2
BivProm1
DNase1
znf2
znf1
TxEx4
TxEx3
TxEx2
TxEx1
Tx8
Tx7
Tx6
Tx5
Tx4
Tx3
Tx2
Tx1
TxWk2
TxWk1
TxEnh8
TxEnh7
TxEnh6
TxEnh5
TxEnh4
TxEnh3
TxEnh2
TxEnh1
EnhA20
EnhA19
EnhA18
EnhA17
EnhA16
EnhA15
EnhA14
EnhA13
EnhA12
EnhA11
EnhA10
EnhA9
EnhA8
EnhA7
EnhA6
EnhA5
EnhA4
EnhA3
EnhA2
EnhA1
EnhWk8
EnhWk7
EnhWk6
EnhWk5
EnhWk4
EnhWk3
EnhWk2
EnhWk1
Acet8
Acet7
Acet6
Acet5
Acet4
Acet3
Acet2
Acet1
ReprPC9
ReprPC8
ReprPC7
ReprPC6
ReprPC5
ReprPC4
ReprPC3
ReprPC2
ReprPC1
HET9
HET8
HET7
HET6
HET5
HET4
HET3
HET2
HET1
Quies5
Quies4
Quies3
Quies2
Quies1
GapArtf3
GapArtf2
GapArtf1

Supplementary Fig. 6: Chromatin states of the mammalian methylation array. (a) The heatmap visualizes the ChromHMM chromatin state annotations of the location of the CpGs on the array (rows) in different human tissues (columns)[5,6]. The colors for the tissue types are shown on left. The colors in the heatmap correspond to 25 human chromatin states as labeled in the right panel and described in Ref. [6]. The rows in the left panel heatmap are ordered by the chromatin state with the maximum frequency across 127 human cell and tissue types. The right panel indicates the distribution of chromatin states in each tissue type represented on the mammalian methylation array. Each cell in the heatmap color codes one of 25 ChromHMM states (legend on the right). In the left panel, the first horizontal red band shows that many CpGs are in transcriptional start sites (TSS states) and promoters across all tissue types. The second, green, band shows that many cytosines are in transcribed regions (Tx) in most tissues and cell types. The orange and yellow color band corresponds to CpGs in enhancers. The purple corresponds to CpGs that are in bivalent chromatin in most cell types. However, the red vertical bands within the horizontal purple band indicate that many of these CpGs are in active promoters specifically in brain or embryonic stem cells. The dark grey horizontal band corresponds to CpGs that are repressed by polycomb repressive complex II (H3K27me3 histone marks). The large white band at the bottom corresponds to CpGs that are located in regions that are quiescent in the plurality of cell types (chromatin state 25). The vertical yellow band in the white horizontal band indicates that many of these CpGs are located in enhancers in brain or embryonic stem cells specifically. The right panel of a, shows that the CpGs on the mammalian array have a similar coverage of chromatin states in all considered tissue types. However, the CpGs have a higher coverage of enhancers in brain cells, neurospheres, and stem cells.
(b) Similar to **Fig. 3b**, but labeling individual states. (**Left**) Distribution of probe overlap with a universal chromatin state annotation resulting from applying the stacked modeling approach of ChromHMM to over 100 human cell/tissue types Ref. [7]. Bars are colored based on their corresponding state group. TSS, transcriptional start site; DNase, DNase I hypersensitivity; znf, zinc finger genes; Het, heterochromatin. (**Right**) The same as left, but showing the fold enrichments of the state relative to a uniform background. The strongest enrichment is seen for some bivalent promoter states. A full characterization of the states can be found in Ref. [7].

Supplementary References

1 Gaidatzis, D., Lerch, A., Hahne, F. & Stadler, M. B. QuasR: quantification and annotation of short reads in R. *Bioinformatics* **31**, 1130-1132, doi:10.1093/bioinformatics/btu781 (2015).

2 Uhlén, M. *et al.* Proteomics. Tissue-based map of the human proteome. *Science* **347**, 1260419, doi:10.1126/science.1260419 (2015).

3 Yue, F. *et al.* A comparative encyclopedia of DNA elements in the mouse genome. *Nature* **515**, 355-364, doi:10.1038/nature13992 (2014).

4 Roadmap Epigenomics, C. *et al.* Integrative analysis of 111 reference human epigenomes. *Nature* **518**, 317-330, doi:10.1038/nature14248 (2015).

5 Ernst, J. & Kellis, M. ChromHMM: automating chromatin-state discovery and characterization. *Nat Meth* **9**, 215-216, doi:10.1038/nmeth.1906 (2012).

6 Ernst, J. & Kellis, M. Large-scale imputation of epigenomic datasets for systematic annotation of diverse human tissues. *Nat Biotech* **33**, 364-376, doi:10.1038/nbt.3157 (2015).

7 Vu, H. & Ernst, J. Universal annotation of the human genome through integration of over a thousand epigenomic datasets. *Genome Biology* **23**, 9, doi:10.1186/s13059-021-02572-z (2022).
