# Supplementary material for: A mammalian methylation array for profiling methylation levels at conserved sequences
Source: Nat Commun. 2022 Feb 10;13:783. doi: 10.1038/s41467-022-28355-z (PMC8831611; doi:10.1038/s41467-022-28355-z)
Supplement: Supplementary file 2 — Description of Additional Supplementary Data files [file 41467_2022_28355_MOESM2_ESM.pdf]

DESCRIPTION OF ADDITIONAL SUPPLEMENTARY DATA FILES

Supplementary Data 1. Human Biomarker CpGs from human Illumina methylation arrays (450K+EPIC). These CpGs are not necessarily conserved in different mammalian species. Rather, they were chosen for the purpose of human biomarker studies.

Supplementary Data 2.
Description of genomes used in gene annotation studies of the CpG probes located on the mammalian array.

Supplementary Data 3.
Orthologous genes in different genomes.

Supplementary Data 4.
The Excel file indicates whether a given CpG is located in a CpG island in the respective species.

Supplementary Data 5. Overlap with constrained sequence elements and ConsHMM conservation state annotations.

Supplementary Data 6. Results from calibration data in 3 species. Pearson correlation between CpGs and the benchmark variable ProportionMethylated in 3 in humans, mice, rats. Genome coordinates have been added.

Supplementary Data 7. Manifest file of the mammalian array.
