# Supplementary material for: A mammalian methylation array for profiling methylation levels at conserved sequences
Source: Nat Commun. 2022 Feb 10;13:783. doi: 10.1038/s41467-022-28355-z (PMC8831611; doi:10.1038/s41467-022-28355-z)
Supplement: Supplementary file 10 — Reporting Summary [file 41467_2022_28355_MOESM10_ESM.pdf]

nature research

Corresponding author(s): Jason Ernst
Steve Horvath

Last updated by author(s): January 17, 2022

Reporting Summary

Nature Research wishes to improve the reproducibility of the work that we publish. This form provides structure for consistency and transparency in reporting. For further information on Nature Research policies, see our Editorial Policies and the Editorial Policy Checklist.

Statistics

For all statistical analyses, confirm that the following items are present in the figure legend, table legend, main text, or Methods section.

n/a	Confirmed	
☐	☒	The exact sample size (*n*) for each experimental group/condition, given as a discrete number and unit of measurement
☐	☒	A statement on whether measurements were taken from distinct samples or whether the same sample was measured repeatedly
☐	☒	The statistical test(s) used AND whether they are one- or two-sided<br>*Only common tests should be described solely by name; describe more complex techniques in the Methods section.*
☐	☒	A description of all covariates tested
☐	☒	A description of any assumptions or corrections, such as tests of normality and adjustment for multiple comparisons
☐	☒	A full description of the statistical parameters including central tendency (e.g. means) or other basic estimates (e.g. regression coefficient) AND variation (e.g. standard deviation) or associated estimates of uncertainty (e.g. confidence intervals)
☐	☒	For null hypothesis testing, the test statistic (e.g. *F*, *t*, *r*) with confidence intervals, effect sizes, degrees of freedom and *P* value noted<br>*Give P values as exact values whenever suitable.*
☒	☐	For Bayesian analysis, information on the choice of priors and Markov chain Monte Carlo settings
☒	☐	For hierarchical and complex designs, identification of the appropriate level for tests and full reporting of outcomes
☐	☒	Estimates of effect sizes (e.g. Cohen's *d*, Pearson's *r*), indicating how they were calculated

Our web collection on statistics for biologists contains articles on many of the points above.

Software and code

Policy information about availability of computer code

Data collection	R_4.0.3: Programming language for statistical computing<br>R_sesame_1.3.0: Normalize Illumina Infinium DNA methylation array data<br>The chip manifest file and genome annotations of the CpGs can be found on Github<br>https://github.com/shorvath/MammalianMethylationConsortium/tree/v1.0.0<br>A vignette on using the mammalian methylation array with SeSAMe is available from<br>https://bioconductor.org/packages/release/bioc/vignettes/sesame/inst/doc/mammal.html<br>The calibration data generated in this study have been deposited in the Gene Expression Omnibus data base under accession codes GSE174567 [https://www.ncbi.nlm.nih.gov/geo/query/acc.cgi?acc=GSE174567] and GSE174568 [https://www.ncbi.nlm.nih.gov/geo/query/acc.cgi?acc=GSE174568]. The bat methylation data are available under accession code GSE164127 [https://www.ncbi.nlm.nih.gov/geo/query/acc.cgi?acc=GSE164127].
Data analysis	Bedtools_2.25.0: Evaluate overlap between probe sets and CpG island status<br>BSBolt_1.4.8: bisulfite sequencing analysis platform<br>eFORGE_V2.0: cell type specific signal in epigenomic data<br>bedTools 2.30.0<br>R_4.0.3: Programming language for statistical computing<br>R_samtools_2.6.0: Utilities for manipulating sequence alignments<br>R_rGREAT_1.22.0: Enrichment analysis using Genomic Regions Enrichment of Annotations Tool<br>R_TissueEnrich_3.14.Tissue-specific gene enrichment analysis<br>R_stats_4.0.3: R base package for fitting si mple linear regressions and computing Pearson's correlations<br>gCluster (v.2.0.2): CpG island annotation<br>R_minfi 1.40.0

R_Chipseeker_1.8.6: Determine distance of CpG to nearest transcription start site
R_QuasR_1.30.0: Align probe sequences to genome assembly
Galaxy server with the default settings of bwa-meth (v0.2.2) and MethylDackel packages (version 0.61)
Biomart Ensembl data base and liftOver, release 105
Annotation database phastCons (version hg19_20110909)

For manuscripts utilizing custom algorithms or software that are central to the research but not yet described in published literature, software must be made available to editors and reviewers. We strongly encourage code deposition in a community repository (e.g. GitHub). See the Nature Research guidelines for submitting code & software for further information.

Data

Policy information about availability of data

All manuscripts must include a data availability statement. This statement should provide the following information, where applicable:

- Accession codes, unique identifiers, or web links for publicly available datasets
- A list of figures that have associated raw data
- A description of any restrictions on data availability

The chip manifest file and genome annotations of the CpGs can be found on Github https://github.com/shorvath/MammalianMethylationConsortium/tree/v1.0.0. The calibration data generated in this study have been deposited in the Gene Expression Omnibus data base under accession codes GSE174567 [https://www.ncbi.nlm.nih.gov/geo/query/acc.cgi?acc=GSE174567] and GSE174568 [https://www.ncbi.nlm.nih.gov/geo/query/acc.cgi?acc=GSE174568]. The bat methylation data are available under accession code GSE164127 [https://www.ncbi.nlm.nih.gov/geo/query/acc.cgi?acc=GSE164127]. The horse array data (Horvath et al., 2022) are available under accession code GSE174767 [https://www.ncbi.nlm.nih.gov/geo/query/acc.cgi?acc=GSE174767]. The Reduced Representation Bisulfite Sequencing from horses (Ząbek et al., 2019) can be downloaded from the SRA database under bioproject No. PRJNA517684 [https://www.ncbi.nlm.nih.gov/bioproject/PRJNA517684/]. The Whole Genome Bisulfite Sequencing data from cattle (Zhou et al., 2020;Liu et al., 2020) can be downloaded under accession code GSE147087 [https://www.ncbi.nlm.nih.gov/geo/query/acc.cgi?acc=GSE147087]. The whole genome bisulfite sequencing data from 37 different tissue types can be downloaded from the Roadmap Epigenomics Consortium [http://egg2.wustl.edu/roadmap/data/byDataType/dnamethylation/WGBS/FractionalMethylation.tar.gz]. We used genome annotations from ENSEMBL https://www.ensembl.org/index.html. The human-mouse LECIF score (Kwon and Ernst, 2021) can be downloaded from [https://github.com/ernstlab/LECIF/]. The universal ChromHMM chromatin state annotations can be downloaded from [https://github.com/ernstlab/full_stack_ChromHMM_annotations] (Vu and Ernst, 2022). The per cell or tissue type specific chromatin state annotations in human can be downloaded from [https://egg2.wustl.edu/roadmap/data/byFileType/chromhmmSegmentations/ChmmModels/imputed12marks/jointModel/final/] (Ernst and Kellis, 2015). The ConsHMM conservation state annotations can be downloaded from [https://github.com/ernstlab/ConsHMM/] (Arneson and Ernst et al., 2019). The constrained element annotations can be downloaded from [http://mendel.stanford.edu/SidowLab/downloads/gerp] (GERP++) (Davydov et al., 2010), [https://www.broadinstitute.org/mammals-models/29-mammals-project-supplementary-info] (SiPhy-omega and SiPhy-omega) (Garber et al., 2009), and [https://genome.ucsc.edu/cgi-bin/hgTables] (PhastCons) (Siepel et al., 2005). The cattle data generated on the mammalian array were not generated for this study. These data are presented in another article (Kordowitzki et al., 2021) and can be requested from SH. The mammalian methylation array (HorvathMammalMethylChip40) is registered at the NCBI Gene Expression Omnibus (GEO) as platform GPL28271. The mammalian methylation array can be purchased from the non-profit Epigenetic Clock Development Foundation (https://clockfoundation.org/). Annotations of the array can be found in Supplementary Data 7.

Field-specific reporting

Please select the one below that is the best fit for your research. If you are not sure, read the appropriate sections before making your selection.

☐ Life sciences ☐ Behavioural & social sciences ☒ Ecological, evolutionary & environmental sciences

For a reference copy of the document with all sections, see nature.com/documents/nr-reporting-summary-flat.pdf

Ecological, evolutionary & environmental sciences study design

All studies must disclose on these points even when the disclosure is negative.

Study description	Infinium methylation arrays are not available for the vast majority of non-human mammals. Moreover, even if species-specific arrays were available, probe differences between them would confound cross-species comparisons. To address these challenges, we developed the mammalian methylation array, a single custom array that measures 36k CpGs that are well conserved across mammalian species. We designed a set of probes on the array that can tolerate specific cross-species mutations. We annotate the array in over 200 species and report CpG island status and chromatin states in select species. Calibration experiments demonstrate the high fidelity in humans, rats, and mice. The mammalian methylation array has several strengths: it applies to all mammalian species even those that have not yet been sequenced, it provides deep coverage of conserved cytosines facilitating the development epigenetic biomarkers, and it increases the probability that biological insights gained in one species will translate to others.
Research sample	The paper uses calibration/titration data for evaluating the fidelity of the array platform.<br>The DNA samples from each species were enzymatically manipulated so that they would exhibit 0%, 25%, 50%, 75% and 100% percent methylation at each CpG location, respectively. The variable “ProportionMethylated” (with ordinal values 0, 0.25, 0.5, 0.75, 1) can be interpreted as a benchmark for each CpG that maps to the respective genome. Thus, the DNA methylation levels of each CpG are expected to have a high positive correlation with ProportionMethylated across the arrays measurement from a given species. The mammalian array was applied to synthetic DNA data from 3 species: human (n=10 mammalian arrays, 2 per methylation level), mouse (n=20, 4 per methylation level), and rat (n=15, 3 per methylation level). Similarly, the human EPIC array was applied to calibration data from mouse (n=15 EPIC arrays, 3 per methylation level) and rat (n=10, 2 per methylation level). The EPIC array data were normalized using the noob method (R function preprocessNoob in minfi).<br>The sample size was informed by statistical power studies for the Pearson correlation based on Pearson correlation (Fisher Z transformation). N=9 samples provide 90% power to detect a correlation of 0.9 at an uncorrected two sided p value of 0.05. At a

	more stringent significance level of alpha=0.0005, N=13 samples provide the same power of 90%.
Sampling strategy	Our experimental study (calibration study) did not involve sampling. Rather, we analyzed all samples that were experimentally generated.
Data collection	We used engineered DNA at different methylation levels to assess the mammalian methylation array's performance and compare it with the human-based EPIC methylation array. The engineered DNA was treated with bisulfite. Then, the methylation profiles were obtained by hybridizing labeled DNA to the mammalian methylation array (HorvathMammalMethylChip40) or EPIC array and scanning with an Illumina iScan at the UCLA Neuroscience Genomics Core.
Timing and spatial scale	The calibration data did not have a time or spacial scale. Rather, we analyzed artificial DNA that was generated in the lab.
Data exclusions	None of the data were excluded.
Reproducibility	Our titration data involve at least 2 replicate per species. We took several measures to make sure the results from the mammalian methylation array is reliable and reproducible. First, we included degenerate bases in the probe design to tolerate cross-species genomic mutations in the probe hybridization site. However, we used stringent alignment criteria to ensure no mismatch in the target CpG location. Second, we used calibration-engineered DNA from mice, rats, and humans to identify the high-quality probes that can linearly measure all ranges of 0-100% methylation levels.
Randomization	The calibration study did not involve an intervention, i.e. randomization was not relevant.
Blinding	Blinding was not relevant to our calibration study since knowledge of the benchmark measure was essential for the analysis.

Did the study involve field work? ☐ Yes ☒ No

Reporting for specific materials, systems and methods

We require information from authors about some types of materials, experimental systems and methods used in many studies. Here, indicate whether each material, system or method listed is relevant to your study. If you are not sure if a list item applies to your research, read the appropriate section before selecting a response.

Materials & experimental systems

n/a	Involved in the study
☒	☐ Antibodies
☒	☐ Eukaryotic cell lines
☒	☐ Palaeontology and archaeology
☒	☐ Animals and other organisms
☒	☐ Human research participants
☒	☐ Clinical data
☒	☐ Dual use research of concern

Methods

n/a	Involved in the study
☒	☐ ChIP-seq
☒	☐ Flow cytometry
☒	☐ MRI-based neuroimaging
